# Effectiveness of digital pain management for older adults with musculoskeletal pain: systematic review with meta-analysis

**DOI:** 10.3389/fpain.2025.1657014

**Published:** 2025-09-17

**Authors:** Anabela G. Silva, Ana J. Santos, Rosa Andias, Nelson P. Rocha

**Affiliations:** ^1^ RISE-Health, School of Health Sciences, University of Aveiro, Aveiro, Portugal; ^2^ RISE-Health, Department of Education and Psychology, University of Aveiro, Aveiro, Portugal; ^3^ RISE-Health, University of Aveiro, Aveiro, Portugal; ^4^ Department of Medical Sciences, Institute of Electronics and Informatics Engineering of Aveiro, University of Aveiro, Aveiro, Portugal

**Keywords:** pain, older adults, digital health, telerehabilitation, mobile health

## Abstract

**Introduction:**

Musculoskeletal pain is highly prevalent among older adults and a leading cause of disability. Digital health promises to deliver timely and quality care, but existing reviews fail to be specific for older adults, focus on a single type of technology or a single body site, and do not provide an integrated overview of the effectiveness of current digital interventions. This systematic review with meta-analysis (Prospero ID: CRD42024549668) aimed to assess the effectiveness of digital interventions for pain management in reducing pain intensity and self-reported disability in older adults with musculoskeletal pain.

**Methods:**

We searched PubMed, Web of Science, Scopus, and Academic Search Complete from inception to April 2025; extracted data on participants, interventions, and primary (pain intensity and self-reported disability) and secondary outcomes (performance, pain-related psychological variables, and adverse events).

**Results:**

Thirty-six RCTs were included (*n* = 4,041). Compared to other active interventions, older adults who received digital pain management reported lower pain intensity (SMD = −0.23, 95%CI = −0.37;−0.09) and lower self-reported disability (SMD = −0.22, 95%CI = −0.39;−0.04) at post-intervention. The effect was maintained at 6 months for pain intensity (SMD = −0.20; 95%CI = −0.38;−0.03), but not for disability (SMD = 0.13, 95%CI = −0.38;0.63). The certainty of evidence was low or very low, and heterogeneity was low to substantial. Most studies included domains judged as high risk of bias.

**Discussion:**

The evidence is very uncertain on the effect of digital interventions on pain intensity and disability. They may decrease pain intensity and disability similarly to other interventions, but more research is needed to investigate the effect of digital interventions and identify key aspects that maximise the intervention.

**Systematic Review Registration:**

https://www.crd.york.ac.uk/PROSPERO/view/CRD42024549668, PROSPERO CRD42024549668.

## Introduction

Musculoskeletal pain affects more than 60% of older adults (1), with the low back, the hip, and the knee being the most common painful body sites (2). Most older adults report pain that is often or always present, is of at least moderate intensity, and located in three or more body sites (3). Furthermore, musculoskeletal pain is associated with decreased functioning, assessed through self-reported or performance-based measures (3). Pain also negatively impacts psychological well-being, being associated with decreased self-efficacy and increased anxiety (4), fear of movement (5) or catastrophizing, which, in turn, might also negatively impact older adultś ability to be physically active (6), further threatening a healthy and active aging.

Pain is one of the main reasons for healthcare use (7–9), with higher pain intensity and disability being drivers of care seeking (10, 11), burdening the healthcare system (2). Nevertheless, there are inequalities in access to adequate pain treatment, particularly non-pharmacological pain treatment (7, 8), that relate to high costs, remote healthcare centers, and difficulty accessing transportation (12).

Digital health offers the possibility to overcome current barriers to provide quality and timely non-pharmacological pain management interventions to larger numbers of individuals at lower costs (13). Digital health is a broad term that includes all tools and services using information and communication technology to support healthcare (14), including those accessed, for example, by a computer or a mobile phone. Also, digital health allows interaction with the health professional, which can be either synchronous, allowing real-time face-to-face interaction between the patient and the healthcare professional, or asynchronous, allowing the review of the patient's performance or data after the intervention (15). The diversity of digital means and interaction models enables the digital intervention's adjustment to the individual needs, characteristics, and preferences, and facilitates personalization (16), while having the potential to impact its use and effectiveness.

Existing systematic reviews suggest that some types of technology might be effective in reducing pain and improving functioning (17, 18), but fail to be specific for older adults (17), focus on a single type of technology, or are limited to one body site (17, 18) not providing an integrated overview of the effectiveness of digital pain interventions for older adults. Therefore, the primary aim of this systematic review was to assess the effectiveness of digital interventions delivered at a distance for pain management in reducing pain intensity and self-reported disability in older adults with musculoskeletal pain. The secondary aim was to explore the effectiveness of digital interventions targeting pain to improve performance-based measures (e.g., Timed Up and Go, gait velocity, grip strength) and pain-related psychological variables (self-efficacy, fear of movement, catastrophizing, and anxiety) in older adults with musculoskeletal pain. Adverse events were also characterised.

## Methods

This systematic review followed PRISMA guidelines (19) and was registered in PROSPERO (registration number: CRD42024549668).

### Search strategy

Pubmed, Web of Science, Scopus, and Academic Search Complete were searched from inception to the 20th of April 2024 and updated on the 14th of April 2025, using the search terms available in the Supplementary Material 1 - Search strategy. Search results were exported to CADIMA (https://www.cadima.info), and duplicates were identified and deleted. Reference lists of selected studies were checked for further relevant studies.

### Selection of studies

Two authors (combined pairs of two of the four authors) independently screened the titles and abstracts, and the full texts. Discrepancies were resolved by consensus in a meeting with the four authors. At the full-text level, reasons for exclusion were documented. Reference screening was conducted with the support of CADIMA software.

### Eligibility criteria

We included studies with older adults with acute (including post-surgical) or chronic musculoskeletal pain aged 60 and over (mean age of at least 60 years old). Musculoskeletal pain was defined as nociceptive pain that arises as part of a disease process directly affecting bone(s), joint(s), muscle(s), or related soft tissue(s) (20). Conditions usually considered as being musculoskeletal but for which the causes are incompletely understood (primary musculoskeletal pain), such as nonspecific back pain or fibromyalgia and chronic widespread pain, are classified in the CID-11 as primary pain (21) were also included.

Interventions included any pain management asynchronous or synchronous digital intervention delivered at a distance from the clinical center/hospital and constituting the main component of the intervention. This was defined as an intervention delivered via any web-based or online platforms, mobile applications, or virtual reality, accounting for at least 75% of the total intervention. The percentage of the intervention delivered digitally was calculated by dividing the total duration or total number of sessions of the intervention administered digitally by the total duration or total number of sessions of the intervention, respectively, multiplied by 100. Additionally, the participant and the health professional were in separate settings (e.g., the participant at home and the professional in a clinical environment). Studies employing digital interventions delivered by health professionals to participants in clinical settings were excluded as this was not considered to be delivered at a distance, and the potential for contact with healthcare professionals was very high, potentially affecting the effect of the intervention. Also, studies that used digital tools solely for data collection or self-monitoring (e.g., number of steps collected from a wearable sensor or mobile application) were excluded.

Comparisons included usual care, no treatment, waiting-list, a placebo (a digital intervention with limited features), or any non-digital pain management intervention. Studies using an active digital intervention in both arms were excluded, as these studies would not provide data on the beneficial effect of digital interventions.

Primary outcomes were pain intensity and self-reported disability measured using any validated instrument. Disability is usually characterized by both self-reported measures, which assess the individuals' perception of their capability to perform a range of tasks, and performance-based measures that capture how well an individual can perform a task and usually involve the completion or timing of strength, balance, or mobility tasks by an assessor (3, 22). Therefore, performance measures were also included as secondary outcomes. Additional secondary outcomes were: pain-related psychological factors (catastrophizing, fear of movement, self-efficacy, and anxiety) and adverse events. We collected outcome data immediately after treatment (baseline), 6-month follow-up (6 months), and 12-month follow-up.

The type of studies included were randomized controlled trials, as randomized controlled trials are one of the highest-quality trial designs for establishing effectiveness. We excluded other study designs, conference abstracts, dissertations, and papers that were not peer-reviewed.

### Data extraction

A customized Excel form for data extraction was tested in three studies to ensure completeness of headings, clear and consistent coding, and response options, and to train researchers (23). The following data was extracted: authors and year of publication, participants' characteristics (age, sex, clinical condition), outcomes, general characteristics of the intervention (type, duration, frequency), characteristics of the digital intervention (delivered synchronously or asynchronously, type of technology used), personalization features of the digital intervention, adverse events and results. Personalization features were characterized using a previously used approach (24) that identified four possible personalization strategies: (i) goal setting (it involves defining goals considering the patients capabilities and preferences); (ii) adjusting the plan (it involves adjusting the intervention based on the capabilities of the participants and feedback throughout the intervention); (iii) using data-driven approaches (it involves gathering data on participants' health status and integrating those data into personalized interventions); and (iv) motivating behavioural changes (including text messages, reminders, and prompts).

### Risk of bias

We used the Cochrane Risk of Bias tool (Rob-2) to judge the risk of bias (25). The domains covered by the tool (randomisation process, deviations from the intended interventions, missing outcome data, outcome measurement, selection of the reported outcome, and overall bias) were rated as “low,” “some concerns,” or “high” risk of bias. It was administered independently by at least two of the four authors, and by type of outcome (i.e., a separate Rob-2 was filled in for self-reported outcomes and clinical tests/performance measures administered by the clinician/assessor). Discrepancies were resolved by discussion among the four authors till a consensus was reached.

### Grading of evidence

The certainty of evidence was assessed using the Grading of Recommendations Assessment, Development and Evaluation (GRADE) approach and rated as high, moderate, low, or very low (26). Certainty in the meta-analysis results was downgraded for serious study limitations (one level if 25% of participants were from studies classified as high risk and two levels if the percentage was 50% or higher), inconsistency (downgrade one level if heterogeneity was high; *I*^2^≥ 75%), imprecision (downgraded one level if there were fewer than 400 participants in each arm), and publication bias (downgraded one level if there was evidence of publication bias assessed through visual inspection of funnel plots and Egger's test). Indirectness was not used to downgrade evidence as participants, comparisons, and outcomes were all directly relevant.

### Summary of evidence

#### Meta-analysis

Comparisons or outcomes were performed at post-intervention and follow-up (when possible). Meta-analyses were conducted using SPSS (IBM, version 28). When data were not available or suitable (e.g., means not reported) for a meta-analysis, we contacted the corresponding author, requesting the necessary data. When needed data was not directly available, but it was possible to compute from available metrics (e.g., standard errors or confidence intervals to calculate SD), conversion was carried out as specified in the Cochrane Handbook (27). When lower scores of different instruments meant different things, the mean values from one set of studies were multiplied by −1 (27). All meta-analyses were conducted with random-effects models because of heterogeneity in study design and outcome measures across trials. We reported standardized mean differences (SMD) and respective 95% confidence intervals. SMD was interpreted as small (0.2), medium (0.5), and large (0.8). Heterogeneity was assessed using *I*^2^, interpreted as low heterogeneity (0%–40%), moderate heterogeneity (30%–60%), substantial heterogeneity (50%–90%), and considerable heterogeneity (75%–100%) (27). The findings of the meta-analysis were conveyed using the statements suggested by Santesso et al. (28), crossing the effect size and the level of certainty of evidence. Results are presented in graphics and a summary table of the effect sizes for individual studies.

Considering the moderate heterogeneity of the main meta-analyses, we explored subgroup analysis for type of patients (i.e., patients with chronic conditions and patients with post-surgery conditions) and sensitivity analysis isolating studies with asynchronous administration of the digital intervention and no personalization/one personalization strategy.

Lower pain intensity, lower self-perceived functioning scores, and lower performance scores represent better outcomes. When more than one instrument was used to assess the same outcome of interest, we prioritize the outcome reported more consistently across studies.

## Results

**Figure 1:**
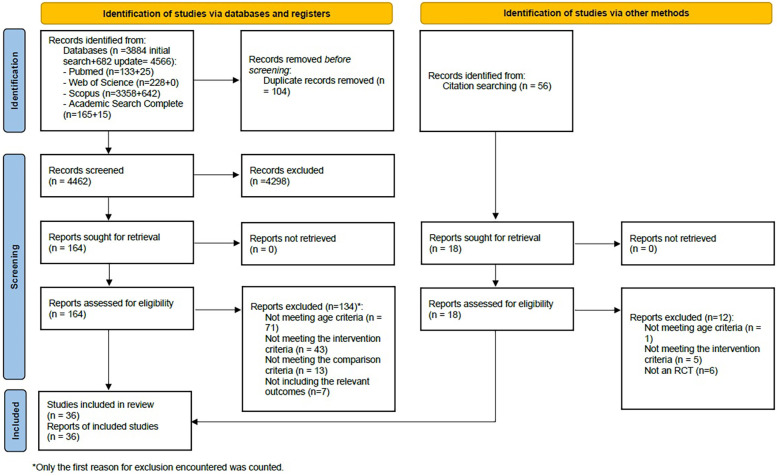
PRISMA 2020 flow diagram.

The search yielded 4,566 records, and after the removal of duplicates, 4,462 unique references were screened, of which 181 full texts were read (164 resulting from database searches and 17 resulting from citation searching). A total of 36 articles, reflecting 36 studies, met the inclusion criteria and were included in this systematic review. The PRISMA flowchart (Figure 1) presents the numbers throughout the selection phases and reasons for exclusion. Corresponding authors of eight articles were contacted, requesting additional information, but only one replied.

### Study characteristics

A total of 36 manuscripts were included that assessed at least one of the main variables of interest (pain intensity or functioning). Included studies represented 4,041 participants, of which 2,270 were females (56.2%). Nineteen studies involved patients with chronic conditions (knee or hip osteoarthritis: *n* = 17; hand osteoarthritis: *n* = 1; low back pain: *n* = 1), while 17 involved patients with acute conditions (hip fracture: *n* = 1) or submitted to surgical procedures (knee or hip arthroplasty: *n* = 13; rotator cuff repair: *n* = 1; carpometacarpal arthroplasty: *n* = 1; spine surgery: *n* = 1). The general characteristics of the studies are presented in Table 1.

**Table 1:** Description of the characteristics of included studies.

References	Number per group	Age	Clinical condition	Control group	Digital intervention		Personalization^a^				A/S
					Description	Duration (D), Frequency (F) Sessions (S)	1	2	3	4	
Weber et al. (56)	D: *n* = 32 C: *n* = 28	*D* = 60.0 ± 6.0 *C* = 64.0 ± 8.0	OA	Usual care; 12-week exercise, physical activity, and education program	App - Join2Move; consists of three modules: graded physical activity, exercise, and education	D: 12 weeks F: 3 days per week	x	x		x	A
Barret et al. (30)	D: *n* = 29 C: *n* = 29	*D* = 61.8 ± 11.1 *C* = 60.2 ± 7.5	Carpometacarpal surgery	Face-to-face sessions; 1x/week, 30 min per session	Video therapy; patients received links to 3 prerecorded exercise videos, each 3 to 4 min long	D: 8 weeks					A
Moutzouri et al. (57)	D: *n* = 22 C: *n* = 22	*D* = 65.1 ± 5.3 *C* = 63.5 ± 5.6	Knee OA	Usual care; General web-based information and advice for OA and encouragement to follow outdoor physical activity 5 times/week and access to the same website as the experimental group, but only to the general recommendations	Blended web-based rehabilitation including a web-based structured video exercise program, OA disease consultatory video sessions, and encouragement to follow an outdoor physical activity walk journey	D: 6 weeks F:3/5 x/week					A
Master et al. (58)	D: *n* = 8 C: *n* = 8	*D* = 65.4 ± 15.7 *C* = 63.0 ± 11.0	Spine surgery	Usual care; this included lifting restrictions, advice to stay active, and oral analgesics as needed. Postoperative physical therapist referral was at the discretion of the surgeon	Zoom; a physical activity intervention including motivational interviewing and SMART goal setting; participants received a Fitbit and a folder that included an introduction to their therapist and the program, a daily step goal tracking sheet, and weekly walking goal	D: 8 weeks F: weekly	x	x	x	x	S
Zhao et al. (29)	D: *n* = 50 C: *n* = 50	*D* = 65 (52–79) *C* = 65 (56–79)	Knee arthroplasty	Home-based rehabilitation following written instructions. The surgeon provided patients with rehabilitation instructions before discharge from the hospital and a rehabilitation manual on how to perform the same training protocol as in the telerehabilitation group	Vital Health Remote Rehabilitation System that consists of three parts: patient-side app, wearable sensors, and surgeon-side websites. The program includes exercises and education	D: 12 weeks	x	x	x		A
Shim et al. (59)	D: *n* = 27 C: *n* = 27	*D* = 68.3 ± 5.8 *C* = 73.0 ± 4.6	Knee arthroplasty	Brochure-based home exercises according to the standard rehabilitation protocol. Both groups performed the same exercises	Augmented-reality; exercise was performed using an AR-based digital system that allowed movement tracking via camera sensors	D: 12 weeks F: daily (30 min per session)					A
Lee et al. (60)	D: *n* = 15 C: *n* = 16	*D* = 65.6 ± 3.7*C* = 68.3 ± 4.8	Knee OA	No intervention.	An app attached to an external device that assesses ROM before each exercise and provides electrostimulation	D:8 weeks F: 3x/week		x	x		A
Janhunem et al. (61)	D: *n* = 25 C: *n* = 27	*D* = 66.9 ± 3.1 *C* = 66.4 ± 4.5	Knee arthroplasty	Home-based exercise. A standard postoperative home exercise program instructed by a physiotherapist	Exergames played using a motion sensor (Kinect 2.0) connected to a laptop and controlled with a tablet	D: 16 weeks F: several times a day					A
Sánchez-Laulhé et al. (62)	D: *n* = 29 C: *n* = 34	*D* = 62.2 ± 8.8 *C* = 64.3 ± 7.7	Hand OA	Home-based exercises delivered using a paper sheet that included pictures and explanations of exercises and dosage	App home exercise program, delivered with the CareHand mobile app	D: 12 weeks F: 4x/week		x		x	A
Thiengwittayaporn et al. (63)	D: *n* = 42 C: *n* = 40	*D* = 62.2 ± 6.8 *C* = 63.0 ± 9.7	Knee OA	Handouts exercises, which were the same as the intervention group	Mobile app featuring four separate modules: basic knowledge of the disease and symptoms, available treatment options, personalized assessment of the stage of severity, and appropriate exercise instructions	D: 4 weeks					A
Nuevo et al. (64)	D: *n* = 23 C: *n* = 22	*D* = 68.3 ± 5.4 *C* = 68.8 ± 4.4	Knee arthroplasty	Leaflet with exercises.	Telerehabilitation system Rehub composed of a web platform and an inertial motion sensor. Patients received a home training session by a physiotherapist and a ReHub® technician 24 h after hospital discharge. The physiotherapist repeated the visit on the following day and after 2 weeks	D: 4 weeks F: daily		x	x	x	A
Cheng et al. (65)	D: *n* = 19C: *n* = 20	*D* = 75.8 ± 7.2 *C* = 79.0 ± 8.8	Hip fracture	Exercise and caregiver skill pamphlet instead of the app. The same home-based rehabilitation program as the digital group	A mobile app on a smart tablet to assist in performing exercises. Both groups received the same homebased rehabilitation program and caregiver skill information	D: 8 weeks F: daily S: 20 to 30 min				x	A
An et al. (66)	D: *n* = 18 C1: *n* = 17 C2: *n* = 18	*D* = 71.1 ± 3.3 C1 = 70.1 ± 2.4 C2 = 70.4 ± 2.6	Knee OA (preoperative)	C1—education about home exercise safety and 40 min of self-home exercise (30 min, 2 times/day, 5 days/week). Telephone calls once a day. C2 - Usual care, including progress monitoring, discharge destination determination, and simple quadriceps exercise performed several times daily	Telerehabilitation program including exercises (mobility, flexibility, strength, balance). The therapist provided supervision and intervention with real-time visual feedback and verbal cues.	D: 3 weeks F: 2 x/daily S: a total of 30 sessions of 30 min each					S
Fanning et al. (67)	D: *n* = 15 C: *n* = 13	*D* = 70.1 ± 5.4 *C* = 70.3 ± 5.2	OA with obesity and chronic pain	Waiting list	Companion App - MORPH, plus Fitbit wearable activity monitor. A mix of in-person group meetings, individual digital calls, and group digital calls. The meetings aimed at providing didactic content related to dietary behaviour change, increasing physical activity, pain management, and mindfulness. Participants received access to weekly educational podcasts and animated videos that reinforced educational content, and they also received regular messages within a “newsfeed” feature	D: 12 weeks	x	x	x	x	A
Doiron-Cadrin et al. (68)	D: *n* = 12 C1: *n* = 11 C2: *n* = 11	*D* = 69.9 ± 9.1 C1 = 61.3 ± 8.1 C2 = 66.7 ± 9.2	Hip and knee OA	C1: face-to-face intervention; C2: provided with the hospital's usual documentation before joint arthroplasty, consisting of information regarding the pre- and post-surgery course and medication use.	Web-based platform; subjects in the tele-prehabilitation group received the same exercise program and advice as C1 through an internet-based telecommunication mobile application after initial assessment.	D: 12 weeks F: 2 supervised sessions/week + repeat on the other days of the week					S
Pronk et al. (69)	D: *n* = 38 C: *n* = 33	*D* = 62.6 ± 7.0 *C* = 64.6 ± 7.5	Knee arthroplasty	Usual care including pre-, peri-, and postoperative pain medication, participated in group information meetings, received an information booklet, and could contact the clinic at any time.	Mobile app – PainCoach + usual care; the app gave the same advice as that during usual care and allowed patients to input their pain level whenever they wanted until day 14 after surgery. Based on the patient's input and number of days after surgery, the app provided advice on pain medication use, physiotherapy exercises including videos, use of ice or heat packs, rest, and immobilization of the operated leg, and when to call the clinic.	D: 4 weeks		x			A
Pelle et al. (70)	D: *n* = 214 C: *n* = 213	*D* = 62.1 ± 7.7 *C* = 62.1 ± 7.0	Knee and hip OA	Usual care defined as non-standardized care initiated by the participant (self-medication, self-referral) to non-medical professionals or initiated by the general practitioner (after consultation initiated by the participant).	The dr. Bart app, which invites users to select pre-formulated goals (i.e., “tiny habits”) and triggers to a healthier lifestyle. The pre-formulated goals are based on four themes: education regarding OA and its treatment, the benefits of a healthy lifestyle, physical activity, vitality, and nutrition	Not specified	x	x		x	A
Bettger et al. (31)	D: *n* = 151 C: *n* = 153	EG = 65.4 ± 7.7 CG = 65.1 ± 9.2	Knee arthroplasty	Usual care including the patients’ care team's recommendations for all preoperative and postoperative medical and rehabilitative care.	The Virtual Exercise Rehabilitation Assistant (VERA), which uses 3D tracking technology to quantify pose and motion, an avatar (digitally simulated coach) to demonstrate and guide activity, visual and audible instructions and immediate feedback on exercise quality, and a virtual video connection for synchronous telehealth visits with an assigned intervention telehealth physical therapist. Individualized prescribed therapy regimens were electronically programmed.	D & F: were unrestricted		x			A + S
Zadro et al. (71)	D: *n* = 30 C: *n* = 30	*D* = 68.8 ± 5.5 *C* = 67.8 ± 6.0	Low back pain	Usual activities	Video-game; participants engaged in an unsupervised preselected home-based exercise program for 8 weeks using a Nintendo Wii U console; including flexibility, body weight resistance, and aerobic exercises	D: 8 weeks F: 3x/week S: 60 min				x	A
Kloek et al. (72)	D: *n* = 109 C: *n* = 99	*D* = 63.8 ± 8.5 *C* = 62.3 ± 8.9	Hip or knee OA	Face-to-face physiotherapy according to the Dutch OA guideline, which recommends: information, physical exercise, and strength and stability exercises (i.e., the same elements as in the intervention group).	Web based platform; including a combination of about 5 face-to-face sessions with a physical therapist and an online application focusing on behavioural graded activity/exercises (grade activity, strength, and stability) and information on OA, pain management, and weight management. Weekly automatic emails informed and reminded patients about new assignments and content.	D: 12 weeks	x			x	S + A
Bennell et al. (32)	D: *n* = 74 C: *n* = 74	*D* = 60.8 ± 6.5 *C* = 61.5 ± 7.6	Knee pain	Internet-based educational material, similar to the intervention group.	PainCOACH + 7 Skype sessions with a physiotherapist; Included educational modules about exercise and physical activity, pain management, emotions, healthy eating, complementary therapies, and medications and practice pain-coping skills daily.	D: 12 weeks F: daily		x		x	A + S
Rini et al. (73)	D: *n* = 58 C: *n* = 55	*D* = 68.5 ± 7.7 *C* = 66.7 ± 11.0	Knee or hip OA	No intervention	PainCOACH program, which translates an in-person pain coping skills training protocol, including eight modules completed without therapist contact, at the rate of one per week. Each module provided interactive training in a cognitive or behavioral pain coping skill. Participants were asked to practice each new skill after learning it, and their completion of and experiences were reviewed at the beginning of the next module.	D: 8 weeks S: each module took 35–45 min to complete	x	x		x	A
Bossen et al. (74)	D: *n* = 10° C: *n* = 99	*D* = 61.0 ± 5.9 *C* = 63.0 ± 5.4	Knee or hip OA	Waiting list	Web-based platform Join2move, which incorporates a baseline test, goal setting, time-contingent PA objectives, and text messages to promote PA. It is a fully automated Web-based intervention that contains automatic functions (web-based text messaging and automatic emails). The patient's favourite recreational activity is gradually increased in a time-contingent way.	D: 9 weeks	x	x		x	A
Gohir et al. (75)	D: *n* = 67 C: *n* = 79	*D* = 65.2 ± 9.7 *C* = 68.0 ± 8.6	Knee or hip OA	Exercise and information following the NICE guidelines. Participants in the usual care group could continue to seek health care input for their knee pain as required during the duration of their study participation.	Mobile app, with structured exercise and OA disease information. Adherence was encouraged by daily emails or smartphone notifications, or by the physiotherapist *via* asynchronous chat or telephone during the study period.	D: 6 weeks	x			x	A
Duong et al. (33)	D: *n* = 51 C: *n* = 51	*D* = 69.0 ± 8.0 *C* = 66.8 ± 6.1	Knee arthroplasty	Usual care	Usual care + 6-month digital technology package consisting of an exercise program delivered through an app (PhysiApp), a Fitbit tracker (step, sleep, and active hours monitoring), and fortnightly health coaching for 3 months. Participants also received motivational text messages on alternating weeks, updating them about their progress	D: 24 weeks S: each session lasted 30 to 45 min	x			x	A + S
Charlton et al. (76)	D: *n* = 10; C: *n* = 10	*D* = 66.8 ± 7.2 *C* = 67.9 ± 3.2	Knee OA	No intervention/ waiting list	Five weekly videoconference appointments involving 15–20-minute Zoom meetings to support participants as they integrated the gait modification into their at-home walking. Behaviour change techniques were used, which involved discussing the benefits of the intervention, identifying barriers and potential solutions, setting weekly goals, and self-monitoring with the walking diary	D: 6 weeks	x				S
Moffet et al. (77)	D: *n* = 104 C: *n* = 101	*E* = 65.0 ± 8.0 CG = 67.0 ± 8.0	Knee arthroplasty	Standard rehabilitation; home visits by the physiotherapist.	Videoconference; the intervention included an assessment before and after exercise, supervised exercises (mobility, strengthening, function, and balance), exercises to perform on days without supervised sessions, and advice concerning pain control, walking aids, and the return to activities	D: 8 weeks S: 16 sessions of 45 to 60 min		x			S
Osterloh et al. (78)	D: *n* = 13 C: *n* = 13	*D* = 65.7 ± 2.8 *C* = 64.0 ± 2.8	Hip and knee arthroplasty	Usual care. Outpatient therapy (a mean of 21 sessions per participant).	Video-based rehabilitation tool—YOLii - twice a week (30 min each session) from the 1st to the 6th postoperative month. Participants received standardized group therapy with the video-based rehabilitation tool YOLii. a pre-selection of exercises was suggested to the physiotherapist, which was then individually adapted to the patient's needs. This allows supervision of up to five patients simultaneously by one therapist.	D: 20 weeks F: 2x/week S: 30 min each, a mean of 39 sessions per participant		x			S
Timmers et al. (79)	D: *n* = 114 C: *n* = 99	*D* = 64.7 ± 7.6 *C* = 65.6 ± 7.9	Knee arthroplasty	Sham. The Patient Journey App; The control group only received basic information about the recovery process, about two times per week	The Patient Journey App; during the 28 days after discharge, every patient received over 30 notifications with supporting information, related to topics such as pain, physiotherapy exercises, wound care, and daily self-care activities	D: 4 weeks S: 30 notifications		x		x	A
Crawford et al. (34)	D: *n* = 208 C: *n* = 244	*D* = 62.8 ± X; *C* = 64.0 ± 8.8	Knee arthroplasty	Standard of care rehabilitation. All participating sites engaged in rapid recovery pathways and nearly universally prescribed physiotherapy following surgery, three times per week. Protocols and instructional and educational content were site-specific.	Smartphone-based telerehabilitation consisting of a patient app on both the iPhone and Apple Watch, as well as a web portal for clinicians that allows them to view details on the patient's engagement, activity levels, patient-reported outcome measures, and messages. Patients were provided preoperative educational content and instructions, along with postoperative educational material and an at-home app-based therapy programme. The app provided patients with reminders to complete their educational and exercise modules.	D: 6 weeks F: 3x/day, six days per week				x	A
Tousignant et al. (80)	D: *n* = 24 C: *n* = 24	*D* = 66.0 ± 10.0 *C* = 66.0 ± 13.0	Knee arthroplasty	Usual home care/outpatient clinic group delivered as usual over about two months; 1 h per session.	Telerehabilitation guided by a physical therapist	D:8 weeks F: 2x/week					S
Kane et al. (81)	EG: *n* = 28 CG: *n* = 30	*D* = 60.6 (39–73) *C* = 59.8 (50–70)	Rotator cuff repair	Face-to-face visits. Patients in the control group were seen by the operating surgeon in the office at 2, 6, and 12 weeks after surgery and received in-person instructions to perform ROM activities.	Telemedicine platform allowing follow-ups with the surgeon at 2, 6, and 12 weeks after surgery and enabled patients to connect with the operative surgeon via webcam using a smartphone, computer, or tablet. Subjects received instructions via mail after the second visit (6 weeks) to begin active-assisted ROM.	Unclear					S
Nelson et al. (82)	D: *n* = 35 C: *n* = 35	*D* = 62.0 ± 9.0 *C* = 67.0 ± 11.0	Hip arthroplasty	Paper-based home exercise programme performed by physiotherapists; The programme was progressed based on the physiotherapists’ assessment during the six-week intervention period.	Two apps; the intervention was identical in content to the control programme, delivery via telerehabilitation technology on an Apple iPadTM. Home exercises were facilitated using an application, and physical therapy sessions were conducted via another app. Compliance was monitored, communication via a messaging feature was possible, and real-time videoconferencing.	D: 2 to 6 weeks		x		x	A + S
Allen et al. (83)	D: *n* = 142 C1: *n* = 140 C2: *n* = 68	*E* = 65.3 ± 11.5 C1 = 65.7 ± 10.3 C2 = 64.3 ± 12.2	Knee OA	C1: Face-to-face physical therapy; participants could receive up to eight one-hour sessions C2: Waiting list	Therapeutic Exercise Resource Center (TERC), a comprehensive web-based system designed to evaluate, prescribe, monitor, and adjust therapeutic exercise programs for patients with knee OA.	D: 48 weeks F: 3x/week for strengthening and stretching exercises; daily for aerobic exercises		x		x	A
Lo et al. (35)	D: *n* = 15 C: *n* = 15	*D* = 63 *C* = 64	Knee OA	Home exercise. A 30-minute health talk led by a physical therapist to explain and demonstrate the home-based exercises that should be performed 5days/per week (30 min per day).	VR iKnee group was instructed to perform lower limb exercises for 12 weeks using an immersive VR platform.	D: 12 weeks F: 5 days/week; S: 30 min per day				X	A
Akgül et al. (84)	D: *n* = 15 C: *n* = 15	*D* = 58.7 ± 5.2 *C* = 62.1 ± 8.6	Knee OA	Standard education + exercises (the same as the intervention group)+smartwatch	1 h face-to-face education + digitally supported walking programme recommendations (WhatsApp) and smartwatches + home exercise programme	D: 8 weeks F: 3x/week, 3 sets of 10 repetitions S: daily	x		x	x	A + S

D, digital intervention group; C, control group; OA, osteoarthritis; PA, physical activity; S, synchronous; A, asynchronous; ROM, range of motion; VR, virtual reality.

^a^ Elements of personalization of the digital intervention: (1) goal setting; (2) adjusting the plan; (3) data-driven approaches; (4) motivating behavioural changes.

The digital intervention was delivered synchronously, via teleconference software, in 8 (22%) studies, and asynchronously, via mobile apps, web-based platforms, video, exergames, or virtual reality, with or without external sensors for data collection, in 22 (61%) studies. In addition, 6 (17%) studies combined a synchronous and an asynchronous component.

The digital intervention was compared against other interventions in 31 (86%) studies, defined as usual care (*n* = 14), face-to-face intervention (*n* = 3), home care (*n* = 5), a brochure with information/exercises (*n* = 4), and general online/app-delivered information (*n* = 2). Also, of the 31 studies, two studies included two comparison groups receiving a combination of usual care, home exercises, or face-to-face care, and another study included one active comparison arm (usual care) and a waiting list control. Most of the studies using a comparator defined as “usual care”, “face-to-face intervention”, and “home care” involved some form of education and exercise. The remaining 5 (14%) of the 36 included studies used a no-intervention/wait-list group as a control.

Regarding personalization strategies, 9 (25%) studies did not include any, 9 (25%) included only 1, and an additional 9 (25%) studies included two. Only 9 (25%) studies used 3 or 4 personalization strategies.

The duration of the digital intervention varied (range: 3 weeks–48 weeks), with 24 (67%) studies reporting 8 or more weeks, 10 (28%) reporting 6 or fewer weeks, and 2 (5%) were unclear.

Of 36 included studies, 32 reported on pain intensity, 34 reported on self-reported disability, and 22 reported on a performance measure. The most commonly used instruments to assess pain intensity were the numeric pain rating scale or the visual analogue scale (*n* = 19, 59%). The most commonly used instruments for self-reported disability were the Western Ontario and McMaster Universities Arthritis Index (WOMAC) or WOMAC function subscale (*n* = 13, 38%), and the Hip or Knee Disability and Osteoarthritis Outcome Score (HOOS or KOOS; *n* = 12, 35%). Performance was assessed using mainly the Timed Up and Go test (*n* = 12; 55%).

### Adverse events

Studies that reported on adverse events *n* = 21 (58%) reported either no adverse/serious events or a similar rate of events between both groups (*n* = 14, 67%). Of these, 7 studies reported more concrete data: a 2% rate of adverse events in each group (29), 1 person in the digital group intervention developed a complex regional pain syndrome (30), a rate of falls of 19.4% in the group receiving the digital intervention against 14.6% in the control group and a mean(±sd) of rehospitalizations in 12 weeks of 0.1 ± 0.3 in the experimental group and of 0.2 ± 0.5 in the control group (31), that more participants in the intervention group (*n* = 22) than the control group (*n* = 3) reported minor, mainly unrelated to the intervention, adverse events (15 and 3 events, respectively) (32), adverse events related to skin irritation due to the bandages used to affix tracking sensors (*n* = 3 out of 51 participants) (33), emergency department visits within 90 days were lower in the digital group intervention when compared to the control group [*n* (%): 16 (8.2) vs. 5 (2.5), *p* < 0.013] (34) and in a study using virtual reality (35), 33% of participants reported cybersickness.

### Risk of bias

**Figure 2:**
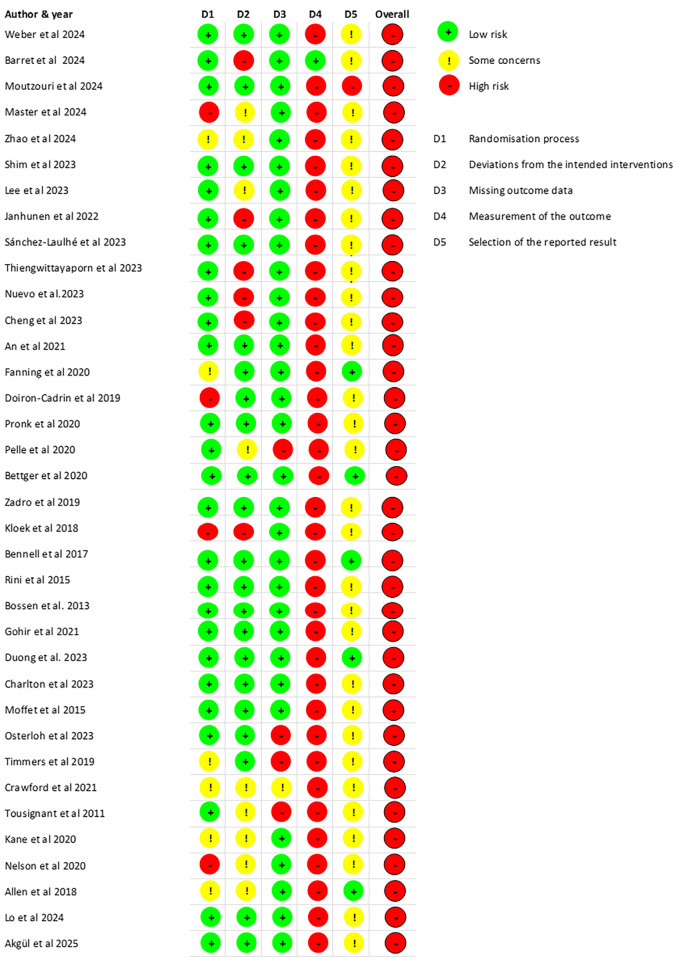
Risk of bias for all studies (pain intensity and self-reported disability—patient-reported outcome measures).

For subjective measurements (pain intensity and self-reported disability), the overall risk of bias was judged as high, due to bias in domain 4 (measurement of the outcome) introduced by lack of blinding. Among the remaining domains, bias was considered low in 26 studies (72%) for domain 1 (randomization process), 21 (58%) for domain 2 (deviations from intended interventions), 86% for domain 3 (missing outcome data) and 5 (14%) for domain 5 (selection of the reported results). For performance measurements and the 22 studies that reported on them, the overall risk of bias was judged as some concerns for 7 studies (32%) and high risk for 15 (68%) studies. Low risk of bias was found for 14 (64%) studies in domain 1, 10 (45%) in domain 2, 19 (86%) in domain 3, 12 (55%) in domain 4, and 3 (14%) in domain 5. The risk of bias for pain intensity and self-reported disability (patient-reported outcomes) is presented in Figure 2. The risk of bias for performance-based measures is presented in Supplementary Material 2.

### Meta-analysis on the effects of the digital intervention

Of the 32 studies that reported pain intensity, 29 provided data that could be included in the meta-analysis (23 compared a digital intervention against another active intervention, and 6 against no intervention). Of the 33 studies that reported self-reported disability, 28 provided data that could be included in the meta-analysis (24 compared a digital intervention against another active intervention, and 4 against no intervention). GRADE tables for all meta-analyses performed are presented in Supplementary Material 3. Additionally, sample size, mean, and standard deviation at post-intervention and follow-up for studies included in the meta-analysis are presented in Supplementary Material 4.

#### Effect of the digital interventions when compared against other forms of treatment on pain intensity and self-reported disability

**Figure 3:**
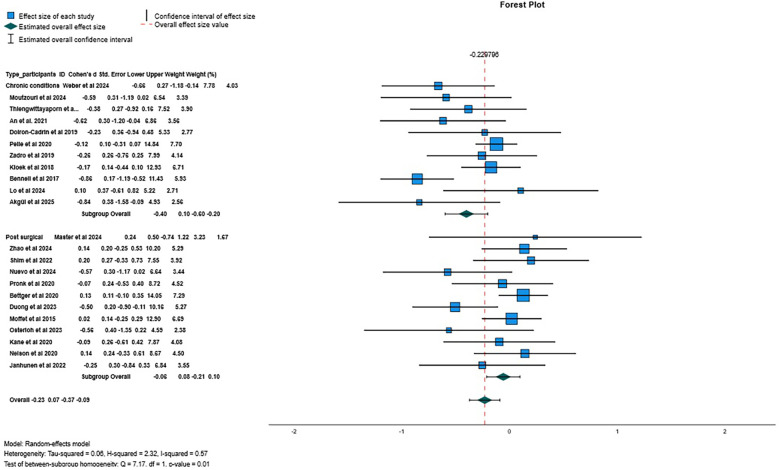
Forest plot for pain intensity when comparing digital interventions against other interventions.

There was low certainty evidence of a small beneficial effect of digital interventions in reducing pain intensity at post-treatment (SMD = −0.23, 95%CI = −0.37 to −0.09, I^2^ 57%, *k* = 23; Figure 3), and very low certainty of evidence that this beneficial effect was maintained at 6 months follow-up (SMD = −0.20; 95%CI = −0.38 to −0.03; *I*^2^ = 0%; *k* = 3). No meta-analysis was possible at 12-month follow-up (*k* = 2).

**Figure 4:**
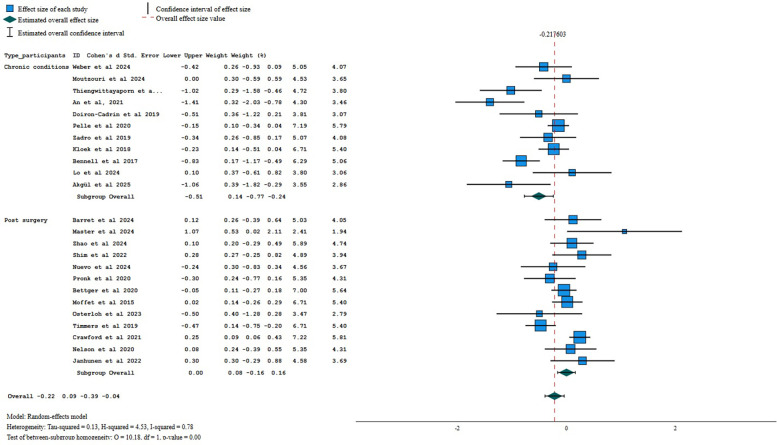
Forest plot for self-reported disability when comparing digital interventions against other interventions.

There was low certainty evidence of a small beneficial effect of digital interventions in reducing disability at post-treatment (SMD = −0.22, 95%CI = −0.39 to −0.04; *I*^2^ 78%, *k* = 24; Figure 4), and very low certainty of evidence that this effect was not maintained at 6-month follow-up (SMD = 0.13, 95%CI = −0.38 to 0.63; *I*^2^ 70%, *k* = 3), neither at 12-month follow-up (SMD = −0.06, 95%CI = −0.23 to 0.11; *I*^2^ = 9%, *k* = 4). Additional flowcharts are presented in Supplementary Material 5. A qualitative description of the results of studies not included in the meta-analyses is presented in Supplementary Material 6.

#### Effect of the digital interventions when compared to no intervention

##### Meta-analysis included only studies with chronic conditions

There was very low certainty of evidence of a small beneficial effect of digital interventions in reducing pain intensity at post-treatment (SMD = −0.24, 95%CI = −0.40 to −0.08, I^2^ 0%, *k* = 6).

There was very low certainty of evidence of no between-group differences at post-treatment for self-reported disability (SMD = −0.09, 95%CI = −0.30 to 0.12, I^2^ 0%, *k* = 4).

No meta-analysis was possible at follow-up due to the small number of studies with the same time-point (*k* < 3).

#### Effect of the digital interventions on performance (secondary outcome)

Of the 22 studies that reported performance, 18 provided data for the meta-analysis (15 compared a digital intervention against another active intervention and 3 against no intervention).

**Figure 5:**
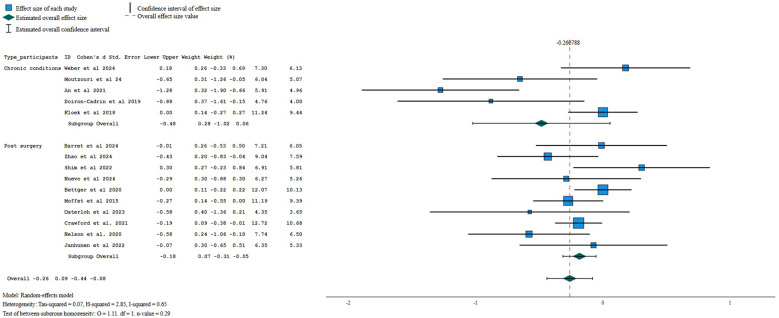
Forest plot for performance when comparing digital interventions against other interventions.

When compared against other interventions, there was very low certainty evidence of a small beneficial effect of digital interventions improving performance at post-treatment (SMD = −0.26, 95%CI = −0.44 to −0.08; *I*^2^ 65%, *k* = 15; Figure 5). No meta-analysis was possible at 6- or 12-month follow-up (*k* < 3 for both follow-up time points).

When compared against no interventions, there was very low certainty of evidence of a small beneficial effect of digital interventions in improving performance at post-treatment for older adults with chronic painful conditions (SMD = −0.49, 95%CI = −0.95 to −0.03, *I*^2^ 0%, *k* = 3).

#### Effect of the digital interventions on psychological variables (secondary outcome)

Six studies assessed at least one psychological variable (self-efficacy, fear of movement, catastrophizing, or anxiety). Meta-analysis was possible only for self-efficacy at post-intervention, when the comparison group was other interventions, and there was very low certainty of evidence of no between-group differences (SMD = 0.39; 95%CI −0.19 to 0.98, I^2^ 87%; *k* = 3).

#### Subgroup and sensitivity analysis for the comparison of digital interventions against other interventions

Given the variability in patients' characteristics, particularly post-operative individuals and patients with chronic conditions, and the moderate heterogeneity identified, we conducted a subgroup analysis based on the type of patients and a sensitivity analysis grouping only studies using one or no personalization strategies, and studies using asynchronous digital interventions. These analyses were only possible when the comparisons were other interventions, due to the small number of studies that used no intervention as a comparison.

Sub-group analysis by type of patients revealed low certainty of evidence of a small beneficial effect of digital interventions when compared to other interventions for pain intensity in participants with chronic conditions (−0.40, 95% CI −0.60 to −0.20; *p* = 0.00; *I*^2^ 53%, *k* = 11) but not for patients who underwent a surgery (−0.06, 95% CI −0.21 to 0.10; *p* = 0.00, *I*^2^ 30%; *k* = 12). Sensitivity analysis, for pain intensity showed a small beneficial effect of asynchronous digital interventions (−0.18, 95% CI −0.34 to −0.03, *p* = 0.02, *I*^2^ 18%, *k* = 11; low certainty of evidence). When aggregating only the studies using one or no personalization strategies, no between-group difference was found (SMD = −0.14, 95% CI −0.31 to 0.02, *p* = 0.20; *I*^2^ = 2%; *k* = 7; low certainty of evidence).

For self-reported disability, sub-group analysis by type of patients revealed low certainty of evidence of a medium beneficial effect of digital interventions when compared to other interventions for patients with chronic conditions (−0.51, 95% CI −0.77 to −0.24; *I*^2^ 74%, *k* = 11), but no difference between interventions for post-operative patients (SMD = 0.00; 95% CI −0.16 to 0.16; *I*^2^ = 54%; *k* = 13). Sensitivity analysis, using only the asynchronous administration of digital interventions, showed no between-group differences (−0.13, 95%=−0.31 to 0.04, *I*^2^ 65%, *k* = 15; low certainty of evidence). When aggregating only the studies using one or no personalization strategies, no between-group differences were found (SMD = −0.18, 95%CI −0.43 to 0.07; *I*^2^ = 79%; *k* = 14; low certainty of evidence).

Sub-group analysis by type of patients revealed very low certainty evidence of a small beneficial effect of digital interventions when compared to other interventions for performance in postoperative patients (−0.18, 95% CI −0.31 to −0.05; *I*^2^ 20%, *k* = 10) but no difference between interventions for patients with chronic conditions (SMD = −0.48; 95% CI −1.02 to 0.06; *I*^2^ 82%; *k* = 5). Sensitivity analysis, using only the asynchronous administration of digital interventions, showed no between-group differences (−0.13, 95%=−0.27 to 0.01, *I*^2^ 14%, *k* = 9; low certainty of evidence). When aggregating only the studies using one or no personalization strategies, a small beneficial effect was found for performance (SMD = −0.30, 95%CI −0.57 to −0.04; *I*^2^ = 76%; *k* = 10; very low certainty of evidence).

Subgroup analysis did not apply when the comparison was no intervention (all studies in the same type of patients), and sensitivity analysis was not possible (*k* < 3).

## Discussion

This review is a comprehensive evaluation of the effectiveness of digital interventions for older adults with pain. Our results suggest that digital interventions may reduce pain intensity and pain disability slightly at post-intervention and compared to other interventions for older adults with painful chronic conditions, but not for older adults with post-surgical/acute conditions. The evidence is very uncertain on the effect of digital interventions at follow-up on pain intensity and disability. The evidence also suggests that digital interventions may improve performance compared to other interventions, but only for older adults with post-surgical conditions. The evidence is very uncertain and scarce about the effect of digital interventions on self-efficacy. This review provides a broad overview of the effectiveness of digital interventions delivered at a distance for older adults, adding to previous reviews that focus on a single body region, clinical condition or intervention (for example, exercise), do not limit studies to those conducted in older adults or do not clarify whether the digital intervention administered at a distance corresponds to the main component of the intervention (36, 37).

Sub-group analysis by type of patients and sensitivity analysis for studies using fewer personalization strategies and delivering the intervention asynchronously showed a decrease in heterogeneity of varying degrees for pain intensity. For self-reported disability and performance, a decrease in heterogeneity was found only in the analyses by type of patients and asynchronous interventions. These findings suggest that type of patients, number of personalization strategies and mode of administration (synchronous vs. asynchronous) partially explain the variability across studies, but its impact on variability depends on the outcome. Furthermore, the sensitivity analysis also suggests that the mode of administration of the digital intervention (synchronous or asynchronous) might impact its effectiveness, as when analysing only the trials with an asynchronous administration of the intervention, the between-group differences for pain disability and performance were no longer present. Similarly, when analysing the trials with no or one personalization strategy, the between-group differences were no longer present for pain intensity and pain disability. Caution should be taken when interpreting the sensitivity analysis, as a few of them included a low number of studies. Nevertheless, these results highlight the importance of reporting the mode of administration (synchronous vs. asynchronous) and the number of personalization strategies in future trials. These factors should also be considered when aggregating data from different trials.

It is unclear why the type of subjects, mode of administration, and interaction strategies impact the effectiveness of digital interventions. Conceivably, there is more room for improvement in patients submitted to a recent surgery, which, adding to the general positive expectations of patients (38), might contribute to improvements that are less dependent on the specific characteristics of the intervention. Older adults value the received support, the ability to establish a continuous care relationship, and human communication when using digital services (39, 40), which might be perceived as less present in asynchronous interventions. Older adults prefer synchronous communication over asynchronous communication, which is believed to improve communication and comprehension (41). The interaction with the physical therapist, even if asynchronous, but frequent (daily), was described as important for support and encouragement. Older adults might have difficulties with technology (41), which can be mitigated by synchronous intervention as the clinician can help solve any issues. Recent studies also showed that a synchronous intervention decreased anxiety and pain intensity in a few body regions (not all) to a greater extent than an asynchronous digital intervention in adults with fibromyalgia (42) and no differences between synchronous and asynchronous digital delivery of exercises for adults with neck pain (42). Different strategies can be used to promote engagement in asynchronous interventions, including providing the possibility of contacting the healthcare professional, using interactive content, building a sense of community with peers through discussion forums or collaborative activities, and providing feedback by healthcare professionals. Whether the relevance of the mode of administration differs across clinical conditions and age ranges requires further investigation.

Personalization, more likely in synchronous and interactive interventions, is also valued by older adults (39). Self-monitoring, self-motivation, goal setting, and personalized feedback are a few of the strategies identified as key for a successful digital intervention (43) and for motivating individuals to behaviour change (44), such as adhering to the digital intervention or performing the recommended exercise. A previous review on the effectiveness of digital health interventions for older adults with cancer also concluded that multiple personalized features were likely to be more effective in improving self-management outcomes (24). Also, a previous systematic review investigating the evidence supporting the use of digital mental health interventions suggested four factors contributing to the success of digital mental health interventions: (1) ease of use; (2) opportunities for social interactions; (3) having human support; and (4) having the digital mental health interventions tailored to the participants’ needs (24). Also, daily email reminders and feedback were considered motivating factors for individuals with osteoarthrosis to perform the exercises (24).

The overall finding of the current review that digital pain interventions may have similar or greater effects on pain intensity and disability than other interventions align well with previous reviews, even though we were more stringent in the inclusion criteria, as the digital component needed to correspond to at least 75% of the intervention for a study to be included in the present review. It has been found that remote exercise programs were not less effective than in-person physical therapy for pain intensity in patients with osteoarthritis (45–47). Telerehabilitation was comparable to conventional in-person rehabilitation in improving clinical outcomes following total knee replacement (48). Despite the diversity of digital solutions used, a subgroup analysis of a previous systematic review indicated no significant difference among the different digital modes of delivery for pain intensity and physical function (47).

Very few studies explored the effect of digital interventions on self-efficacy, catastrophizing, fear of movement, and anxiety. These variables need to be included in future trials evaluating the effectiveness of digital interventions for older adults, considering their impact and relevance on pain intensity and pain-associated disability. Higher self-efficacy is associated with higher outcome expectations (49) and protects against decreased disability and performance at follow-up (49). Higher kinesiophobia is associated with decreased self-reported physical function and performance (50), and both higher catastrophizing and anxiety are associated with higher pain disability (51).

This systematic review also suggests that digital interventions are safe with few non-serious adverse events, most often similar to those occurring in the group receiving other interventions, suggesting that it is safe to use digital interventions with older adults with both chronic and post-surgical painful conditions. However, caution should be taken when interpreting these data and a systematic assessment of serious and non-serious adverse events is recommended in all future trials. More than 40% of included trials did not report on adverse events, and among those that reported on adverse events, the methodology used for their assessment was not always clear.

### Study limitations

The low quality of included studies, as assessed using Rob2, mostly resulted from the inability to blind outcome assessors for self-reported (participant-reported) measures, which are potentially influenced by the participants' knowledge of the intervention received. This led to the downgrading of the evidence when applying the GRADE. The small sample sizes (*n* < 400) of a few meta-analyses (pain intensity at 6-month follow-up when the comparison were other interventions; pain intensity at post-intervention when the comparison was no intervention; self-reported disability at 6 and 12-month follow up when the comparison were other intervention, self-reported disability when the comparison was no intervention; performance for all comparisons, and self-efficacy when the comparison were other interventions), also led to the downgrading of evidence for imprecision when applying the GRADE. These meta-analyses might have lacked sufficient statistical power to detect between-group differences, which was reflected in the certainty of evidence for the effect estimate. The included studies varied in terms of intervention duration (ranging from 3 to 48 weeks) and frequency (from twice a week to unrestricted use). This diversity might have affected results, as a dose-response result might be expected in interventions targeting pain and disability, with a minimal dose of intervention being needed to achieve meaningful improvement (52). Future studies can explore whether a dose-response relationship exists for digital interventions and whether this varies depending on clinical conditions or intervention content. Few studies were included in the follow-up meta-analysis, weakening any conclusion on the medium and long-term effectiveness of digital interventions. Data extraction was performed by a single reviewer and reviewed for correctness and completeness by a second reviewer who was not blind to the data extracted by the first reviewer and could have been unintentionally influenced by it.

### Research and clinical practice recommendations

The apparent safety of digital interventions and the potential for a positive impact on pain and disability cautiously suggest that digital interventions can be used in clinical practice to decrease pain and self-reported disability and improve performance. The choice between a face-to-face intervention and a digital intervention might be left to patients' preferences and ability to safely and correctly use the digital means needed for the intervention. This is particularly relevant as older adults have less access to digital means and lower digital literacy skills than younger groups (53). Therefore, ensuring that the older adult has access to and can use the digital means necessary for the digital intervention is crucial (53), both before and during the intervention. Choosing technology that allows some degree of personalization or adjustment to the individual's needs and preferences, that is inexpensive, and that is simple to use, may facilitate the use of digital means for healthcare by older adults. Furthermore, when choosing the digital intervention, the clinician might want to give preference to interventions allowing for a synchronous component and personalization features.

Future trials, in addition to employing more methodologically robust designs that overcome the limitations identified in this review, can compare digital interventions with different degrees of personalization and the synchronous and asynchronous administration of the same intervention. Furthermore, most existing trials use older adults with knee and hip osteoarthritis or patients who have undergone hip or knee replacement. Therefore, there is a need to investigate the effectiveness of digital interventions for other painful conditions that are prevalent in older adults, such as low back pain (54), pain in the shoulder and foot (55) and also for multiple painful body sites, as the majority of older adults have at least 3 painful body sites (3).

## Conclusion

Our results suggest that digital interventions are at least as good as other interventions at decreasing pain and self-reported disability and improving performance. Furthermore, for older adults with painful chronic conditions, they may reduce pain intensity and pain disability, at post-intervention, slightly more. The evidence is very uncertain on the effect of digital interventions on pain intensity and disability at follow-up, and on the effect of digital interventions on self-efficacy. Further studies are needed to investigate digital pain management for currently under-investigated clinical conditions, such as low back pain and multisite pain, and to investigate which aspects of digital pain management (e.g., interaction) are likely to have a higher impact on the intervention effect.

## Data Availability

The original contributions presented in the study are included in the article/Supplementary Material, further inquiries can be directed to the corresponding author.
