# Supplementary material for: Effectiveness of digital pain management for older adults with musculoskeletal pain: systematic review with meta-analysis
Source: Front Pain Res (Lausanne). 2025 Sep 17;6:1657014. doi: 10.3389/fpain.2025.1657014 (PMC12484128; doi:10.3389/fpain.2025.1657014)
Supplement: Supplementary file 1 [file Supplementaryfile1.docx]

**Supplementary material 1 - Search strategy.**

**Pubmed**

| Older adults  #1 | (("aged"[MeSH Major Topic] OR ("aged"[MeSH Terms] OR "aged"[All Fields] OR ("older"[All Fields] AND "adults"[All Fields]) OR "older adults"[All Fields])) AND("aging"[MeSH Terms] OR "aging"[All Fields] OR "ageing"[All Fields])) OR "elder*"[All Fields] OR ("old"[All Fields] AND "person*"[All Fields]) OR "old person"[MeSH Terms] OR "old adult"[MeSH Terms] OR ("older[All Fields] AND "adult*"[All Fields]) OR ("old"[All Fields] AND "people*"[All Fields]) OR "old people"[MeSH Terms] OR (("aging"[MeSH Terms] OR "aging"[All Fields] OR "ageing"[All Fields]) AND "person*"[MeSH Terms]) OR (("aging"[MeSH Terms] OR "aging"[All Fields] OR "ageing"[All Fields]) AND "person*"[All Fields]) OR (("aging"[MeSH Terms] OR "aging"[All Fields] OR "ageing"[All Fields]) AND "person*"[All Fields]) OR (("aging"[MeSH Terms] OR "aging"[All Fields] OR "ageing"[All Fields]) AND "person*"[MeSH Terms]) OR (("aging"[MeSH Terms] OR "aging"[All Fields] OR "ageing"[All Fields]) AND "adult*"[MeSH Terms]) OR (("aging"[MeSH Terms] OR "aging"[All Fields] OR "ageing"[All Fields]) AND "adult*"[All Fields]) OR (("aging"[MeSH Terms] OR "aging"[All Fields] OR "ageing"[All Fields]) AND "adult*"[All Fields]) OR (("aging"[MeSH Terms] OR "aging"[All Fields] OR "ageing"[All Fields]) AND "adult*"[MeSH Terms]) OR "geriatric*"[MeSH Terms] OR "geriatric*"[All Fields] OR (("older"[All Fields] OR "olders"[All Fields]) AND "population*"[All Fields]) OR (("older"[All Fields] OR "olders"[All Fields]) |
| --- | --- |
| Pain  #2 | ("back pain"[MeSH Terms] OR ("back"[All Fields] AND "pain"[All Fields]) OR "back pain"[All Fields] OR ("neck pain"[MeSH Terms] OR ("neck"[All Fields] AND "pain"[All Fields]) OR "neck pain"[All Fields]) OR ("Spin"[All Fields] AND ("pain"[MeSH Terms] OR "pain"[All Fields])) OR ("chest pain"[MeSH Terms] OR ("chest"[All Fields] AND "pain"[All Fields]) OR "chest pain"[All Fields] OR ("thoracic"[All Fields] AND "pain"[All Fields]) OR "thoracic pain"[All Fields]) OR ("neck pain"[MeSH Terms] OR ("neck"[All Fields] AND "pain"[All Fields]) OR "neck pain"[All Fields] OR ("cervical"[All Fields] AND "pain"[All Fields]) OR "cervical pain"[All Fields]) OR ("low back pain"[MeSH Terms] OR ("low"[All Fields] AND "back"[All Fields] AND "pain"[All Fields]) OR "low back pain"[All Fields] OR ("lumbar"[All Fields] AND "pain"[All Fields]) OR "lumbar pain"[All Fields]) OR ("back pain"[MeSH Terms] OR ("back"[All Fields] AND "pain"[All Fields]) OR "back pain"[All Fields] OR ("back"[All Fields] AND "ache"[All Fields]) OR "back ache"[All Fields]) OR ("low back pain"[MeSH Terms] OR ("low"[All Fields] AND "back"[All Fields] AND "pain"[All Fields]) OR "low back pain"[All Fields]) OR (("knee"[MeSH Terms] OR "knee"[All Fields] OR "knee joint"[MeSH Terms] OR ("knee"[All Fields] AND "joint"[All Fields]) OR "knee joint"[All Fields]) AND ("pain"[MeSH Terms] OR "pain"[All Fields])) OR ("shoulder pain"[MeSH Terms] OR ("shoulder"[All Fields] AND "pain"[All Fields]) OR "shoulder pain"[All Fields]) OR (("elbow"[MeSH Terms] OR "elbow"[All Fields] OR "elbow joint"[MeSH Terms] OR ("elbow"[All Fields] AND "joint"[All Fields]) OR "elbow joint"[All Fields] OR "elbow s"[All Fields] OR "elbows"[All Fields]) AND ("pain"[MeSH Terms] OR "pain"[All Fields])) OR (("wrist"[MeSH Terms] OR "wrist"[All Fields] OR "wrist joint"[MeSH Terms] OR ("wrist"[All Fields] AND "joint"[All Fields]) OR "wrist joint"[All Fields] OR "wrists"[All Fields] OR "wrist s"[All Fields]) AND ("pain"[MeSH Terms] OR "pain"[All Fields])) OR (("hand"[MeSH Terms] OR "hand"[All Fields]) AND ("pain"[MeSH Terms] OR "pain"[All Fields])) OR (("ankle"[MeSH Terms] OR "ankle"[All Fields] OR "ankle joint"[MeSH Terms] OR ("ankle"[All Fields] AND "joint"[All Fields]) OR "ankle joint"[All Fields] OR "ankles"[All Fields] OR "ankle s"[All Fields]) AND ("pain"[MeSH Terms] OR "pain"[All Fields])) OR (("foot"[MeSH Terms] OR "foot"[All Fields]) AND ("pain"[MeSH Terms] OR "pain"[All Fields])) OR (("hip"[MeSH Terms] OR "hip"[All Fields]) AND ("pain"[MeSH Terms] OR "pain"[All Fields])) OR ("musculoskeletal system"[MeSH Terms] OR ("musculoskeletal"[All Fields] AND "system"[All Fields]) OR "musculoskeletal system"[All Fields] OR "musculoskeletal"[All Fields])) |
| Digital intervention  #3 | (("smartphone"[MeSH Terms] OR "smartphone"[All Fields] OR "smartphones"[All Fields] OR "smartphone s"[All Fields] OR ("smartphone"[MeSH Terms] OR "smartphone"[All Fields] OR "smart phone"[All Fields]) OR ("internet"[MeSH Terms] OR "internet"[All Fields] OR "internet s"[All Fields] OR "internets"[All Fields]) OR ("software"[MeSH Terms] OR "software"[All Fields] OR "software s"[All Fields] OR "softwares"[All Fields]) OR ("mobile applications"[MeSH Terms] OR ("mobile"[All Fields] AND "applications"[All Fields]) OR "mobile applications"[All Fields] OR ("mobile"[All Fields] AND "application"[All Fields]) OR "mobile application"[All Fields]) OR ("website"[All Fields] OR "website s"[All Fields] OR "websites"[All Fields]) OR ("webpage"[All Fields] OR "webpages"[All Fields]) OR "computed"[All Fields] OR "computer s"[All Fields] OR "computers"[MeSH Terms] OR "computers"[All Fields] OR "computer"[All Fields] OR ("digital"[All Fields] OR "digitalisation"[All Fields] OR "digitalised"[All Fields] OR "digitalization"[All Fields] OR "digitalize"[All Fields] OR "digitalized"[All Fields] OR "digitalizer"[All Fields] OR "digitalizing"[All Fields] OR "digitally"[All Fields] OR "digitals"[All Fields] OR "digitization"[All Fields] OR "digitizations"[All Fields] OR "digitize"[All Fields] OR "digitized"[All Fields] OR "digitizer"[All Fields] OR "digitizers"[All Fields] OR "digitizes"[All Fields] OR "digitizing"[All Fields]) OR "Online"[All Fields] OR "app"[All Fields] OR "apps"[All Fields] OR ("telemedicine"[MeSH Terms] OR "telemedicine"[All Fields] OR "telemedicine s"[All Fields]) OR ("telerehabilitation"[MeSH Terms] OR "telerehabilitation"[All Fields]) OR "m-health"[All Fields] OR ("mhealth s"[All Fields] OR "telemedicine"[MeSH Terms] OR "telemedicine"[All Fields] OR "mhealth"[All Fields]) OR ("telemedicine"[MeSH Terms] OR "telemedicine"[All Fields] OR "ehealth"[All Fields]) OR "e-health"[All Fields] OR ("telemedicine"[MeSH Terms] OR "telemedicine"[All Fields] OR ("mobile"[All Fields] AND "health"[All Fields]) OR "mobile health"[All Fields])) AND ("technology-based"[All Fields] OR "technologybased"[All Fields] OR "technology-based"[All Fields])) OR ("virtual"[All Fields] OR "virtuality"[All Fields] OR "virtualization"[All Fields] OR "virtualized"[All Fields] OR "virtualizing"[All Fields] OR "virtuals"[All Fields]) OR ("telerehabilitation"[MeSH Terms] OR "telerehabilitation"[All Fields] OR ("telerehabilitation"[MeSH Terms] OR "telerehabilitation"[All Fields] OR ("tele"[All Fields] AND "rehabilitation"[All Fields]) OR "tele rehabilitation"[All Fields]) OR "tele rehabilitation"[All Fields]) OR ("telemedicine"[MeSH Terms] OR "telemedicine"[All Fields] OR "telemedicine s"[All Fields] OR "tele-medicine"[All Fields]) OR (("technology"[MeSH Terms] OR "technology"[All Fields] OR "technologies"[All Fields] OR "technology s"[All Fields]) AND ("based"[All Fields] OR "basing"[All Fields])) OR "technology-based"[All Fields] OR ("virtual"[All Fields] OR "virtuality"[All Fields] OR "virtualization"[All Fields] OR "virtualized"[All Fields] OR "virtualizing"[All Fields] |
| Type of studies (Medline RCT search filter)  #4 | "randomized controlled trial"[Publication Type] OR "controlled clinical trial"[Publication Type] OR "randomized"[Title/Abstract] OR "placebo"[Title/Abstract] OR "randomly"[Title/Abstract] OR "trial"[Title] OR "clinical trials as topic"[MeSH Terms] |
| #5 | #1 AND #2 |
| #6 | #5 AND #3 |
| #7 | #6 AND #4 |

**Web of Science – 20.04.2024 / 228 references**

| Older adults  #1 | **((((((((ALL=("older adult")) OR ALL=("older person")) OR ALL=("older people")) OR ALL=(aging)) OR ALL=(ageing)) OR ALL=(geriatric)) OR ALL=("old adult")) OR ALL=("old person"))** |
| --- | --- |
| Digital intervention  #2 | **((((((((((((((ALL=("digital health")) OR ALL=(digital)) OR ALL=(mhealth)) OR ALL=(m-health)) OR ALL=("mobile health")) OR ALL=(ehealth)) OR ALL=(e-health)) OR ALL=(virtual)) OR ALL=(telemedicine)) OR ALL=(tele-medicine)) OR ALL=(website)) OR ALL=(smartphone)) OR ALL=(telerehabilitation)) OR ALL=(technology-based)) OR ALL=(tele-rehabilitation)** |
| Pain  #3 | pain |
| Type of studies #4 | **((((((ALL=("randomized controlled trial")) OR ALL=("controlled clinical trial")) OR ALL=(randomized)) OR ALL=(placebo)) OR ALL=(randomly)) OR ALL=(trial)) OR ALL=("clinical trial")** |
| #5 | **#1 AND #2 AND #3 AND #4** |

**Academic Search Complete powered by EBSCO – 20.04.2024 / 165 references**

| **Search string** | **("older adults" or elderly or seniors or geriatrics OR “older people” OR “older person” OR aging OR ageing OR “old person” OR “old adult”) (Abstract or authors supplied abstract) AND (Pain) (ALL Tx) AND ("digital health" OR digital OR mhealth OR “m-health” OR "mobile health" OR ehealth” OR “e-health” OR virtual OR telemedicine OR “tele-medicine” OR website OR smartphone OR telerehabilitation OR “technology-based” OR “tele-rehabilitation”) (Abstract or authors supplied abstract) AND (randomly OR "randomized controlled trials" or rtc or "randomised control trials" or "clinical controlled trial" or "clinical trial") (Abstract or authors supplied abstract)** |
| --- | --- |

**Scopus – 20.04.2024 / 228 references 3358**

| Older adults  #1 | ALL(older adults) OR ALL(old adult) OR ALL(older person) OR ALL(old person) OR ALL(older people) OR ALL(aging) OR ALL(ageing) OR ALL(geriatric) |
| --- | --- |
| Digital intervention  #2 | ALL("digital health") OR ALL(digital) OR ALL(mhealth) OR ALL(m-health) OR ALL("mobile health) OR ALL(e-health) OR ALL(e-health) OR ALL(virtual) OR ALL(telemedicine) OR ALL(tele-medicine) OR ALL(website) OR ALL(smartphone) OR ALL(telerehabilitation) OR ALL(tele-rehabilitation) OR ALL(technology-based) |
| Pain  #3 | ALL(pain) |
| Type of studies #4 | TITLE-ABS ("randomized controlled trial") OR ALL ( “controlled clinical trial ") OR ALL(randomized) OR ALL(placebo) OR ALL(randomly) OR ALL(trial) OR ALL(" clinical  trial ) |
| #5 | **#1 AND #2 AND #3 AND #4** |

**Supplementary Material 2 - Risk of bias for performance-based measures.**

**Supplementary Material 3**

**GRADE: Outcome: Pain intensity; Comparison- other interventions.**

| Quality assessment | | | | | | | Summary of findings | | |  |
| --- | --- | --- | --- | --- | --- | --- | --- | --- | --- | --- |
| N. º of studies | Study design | Risk of bias | Inconsistency | Indirectness | Imprecision | Publication bias | Number of patients | | Effect (95%CI) | Quality |
|  |  |  |  |  |  |  | Digital Health | Other interventions |  |  |
| Pain intensity (all studies) | | | | | | | | | | |
| 23 | RCT | Very serious | Not serious | Not serious | Not serious | Undetected | 1132 | 1121 | -0.23  (-0.37; -0.09) | ⨁⨁◯◯ |
| Pain intensity (studies using participants with chronic conditions) | | | | | | | | | | |
| 11 | RCT | Very serious | Not serious | Not serious | Not serious | Undetected | 583 | 573 | -0.40  (-0.60; -0.20) | ⨁⨁◯◯ |
| Pain intensity (studies using participants with post-surgery conditions) | | | | | | | | | | |
| 12 | RCT | Very serious | Not serious | Not serious | Not serious | Undetected | 549 | 548 | -0.06  (-021; 0.10) | ⨁⨁◯◯ |
| Pain intensity (studies using asynchronous administration of digital interventions) | | | | | | | | | | |
| 11 | RCT | Very serious | Not serious | Not serious | Not serious | Undetected | 514 | 485 | -0.18  (-0.34; -0.03) | ⨁⨁◯◯ |
| Pain intensity (studies using no or 1 personalization strategy) | | | | | | | | | | |
| 7 | RCT | Very serious | Not serious | Not serious | Not serious | Undetected | 521 | 526 | -0.14  (-0.31; 0.02) | ⨁⨁◯◯ |
| Pain intensity (6-month follow-up) | | | | | | | | | | |
| 3 | RCT | Very serious | Not serious | Not serious | Serious | Undetected | 257 | 256 | -0.20  (-0.38; -0.03) | ⨁◯◯◯ |

**GRADE: Outcome: Pain intensity; Comparison- no intervention.**

| Quality assessment | | | | | | | Summary of findings | | |  |
| --- | --- | --- | --- | --- | --- | --- | --- | --- | --- | --- |
| N. º of studies | Study design | Risk of bias | Inconsistency | Indirectness | Imprecision | Publication bias | Number of patients | | Effect (95%CI) | Quality |
|  |  |  |  |  |  |  | Digital Health | Other interventions |  |  |
| Pain intensity (all studies; patients with chronic conditions) | | | | | | | | | | |
| 6 | RCT | Very serious | Not serious | Not serious | Serious | Undetected | 340 | 333 | -0.24  (-0.40; -0.08) | ⨁◯◯◯ |

**GRADE: Outcome: Self-reported disability; Comparison- other interventions.**

| Quality assessment | | | | | | | Summary of findings | | |  |
| --- | --- | --- | --- | --- | --- | --- | --- | --- | --- | --- |
| N. º of studies | Study design | Risk of bias | Inconsistency | Indirectness | Imprecision | Publication bias | Number of patients | | Effect (95%CI) | Quality |
|  |  |  |  |  |  |  | Digital Health | Other interventions |  |  |
| Self-reported disability (all studies) | | | | | | | | | | |
| 24 | RCT | Very serious | Not serious (partially explained by sub-group analysis) | Not serious | Not serious | Undetected | 1404 | 1412 | -0.22  (-0.39; -0.04) | ⨁⨁◯◯ |
| Self-reported disability (studies using participants with chronic conditions) | | | | | | | | | | |
| 11 | RCT | Very serious | Not Serious | Not serious | Not serious | Undetected | 583 | 573 | -0.51  (-0.77; -0.24) | ⨁⨁◯◯ |
| Self-reported disability (studies using participants with post-surgery conditions) | | | | | | | | | | |
| 13 | RCT | Very serious | Not serious | Not serious | Not serious | Undetected | 821 | 839 | 0.00  (-0.16; 0.16) | ⨁⨁◯◯ |
| Self-reported disability (studies using asynchronous administration of digital interventions) | | | | | | | | | | |
| 15 | RCT | Very serious | Not serious | Not serious | Not serious | Undetected | 974 | 956 | -0.13  (-0.31; 0.04) | ⨁⨁◯◯ |
| Self-reported disability (studies using no or 1 personalization strategy) | | | | | | | | | | |
| 14 | RCT | Very serious | Serious | Not serious | Not serious | Undetected | 730 | 769 | -0.18  (-0.31; 0.02) | ⨁⨁◯◯ |
| Self-reported disability (6-month follow-up) | | | | | | | | | | |
| 3 | RCT | Very serious | Not serious | Not serious | Serious | Undetected | 257 | 256 | -0.13  (-0.38; 0.63) | ⨁◯◯◯ |
| Self-reported disability (12-month follow-up) | | | | | | | | | | |
| 4 | RCT | Very serious | Not serious | Not serious | Serious | Suspected | 316 | 308 | -0.06 (-0.23; 0.11) | ⨁◯◯◯ |

**GRADE: Outcome: Self-reported disability; Comparison - no intervention.**

| Quality assessment | | | | | | | Summary of findings | | |  |
| --- | --- | --- | --- | --- | --- | --- | --- | --- | --- | --- |
| N. º of studies | Study design | Risk of bias | Inconsistency | Indirectness | Imprecision | Publication bias | Number of patients | | Effect (95%CI) | Quality |
|  |  |  |  |  |  |  | Digital Health | Other interventions |  |  |
| Self-reported disability (all studies; patients with chronic conditions) | | | | | | | | | | |
| 4 | RCT | Very serious | Not serious | Not serious | Serious | Suspected | 180 | 177 | -0.09  (-0.30; 0.12) | ⨁◯◯◯ |

**GRADE: Outcome: Performance; Comparison- other interventions.**

| Quality assessment | | | | | | | Summary of findings | | |  |
| --- | --- | --- | --- | --- | --- | --- | --- | --- | --- | --- |
| N. º of studies | Study design | Risk of bias | Inconsistency | Indirectness | Imprecision | Publication bias | Number of patients | | Effect (95%CI) | Quality |
|  |  |  |  |  |  |  | Digital Health | Other interventions |  |  |
| Performance (all studies) | | | | | | | | | | |
| 15 | RCT | Very serious | Not serious | Not serious | Not serious | Strongly suspected | 854 | 905 | -0.26  (-0.44; -0.08) | ⨁◯◯◯ |
| Performance (studies using participants with chronic conditions) | | | | | | | | | | |
| 5 | RCT | Very serious | Serious | Not serious | Serious | Suspected | 193 | 206 | -0.48  (-1.02; 0.06) | ⨁◯◯◯ |
| Performance (studies using participants with post-surgery conditions) | | | | | | | | | | |
| 10 | RCT | Very serious | Not serious | Not serious | Not serious | Strongly suspected | 661 | 699 | -0.18  (-0.31;-0.05) | ⨁◯◯◯ |
| Performance (studies using asynchronous administration of digital interventions | | | | | | | | | | |
| 9 | RCT | Very serious | Not serious | Not serious | Not serious | Undetected | 521 | 546 | -0.13 (-0.27;0.01) | ⨁⨁◯◯ |
| Performance (studies using no or 1 personalization strategy) | | | | | | | | | | |
| 10 | RCT | Very serious | Serious | Not serious | Not serious | Suspected | 605 | 671 | -0.30 (-0.57;-0.04) | ⨁◯◯◯ |

**GRADE: Outcome: Performance; Comparison - no intervention.**

| Quality assessment | | | | | | | Summary of findings | | |  |
| --- | --- | --- | --- | --- | --- | --- | --- | --- | --- | --- |
| N. º of studies | Study design | Risk of bias | Inconsistency | Indirectness | Imprecision | Publication bias | Number of patients | | Effect (95%CI) | Quality |
|  |  |  |  |  |  |  | Digital Health | Other interventions |  |  |
| Performance (all studies; patients with chronic conditions) | | | | | | | | | | |
| 4 | RCT | Very serious | Not serious | Not serious | Serious | Suspected | 37 | 39 | -0.49  (-0.95; -0.03) | ⨁◯◯◯ |

**GRADE: Outcome: Self-efficacy; Comparison- other interventions.**

| Quality assessment | | | | | | | Summary of findings | | |  |
| --- | --- | --- | --- | --- | --- | --- | --- | --- | --- | --- |
| N. º of studies | Study design | Risk of bias | Inconsistency | Indirectness | Imprecision | Publication bias | Number of patients | | Effect (95%CI) | Quality |
|  |  |  |  |  |  |  | Digital Health | Other interventions |  |  |
| Self-efficacy (all studies) | | | | | | | | | | |
| 3 | RCT | Very serious | Serious | Not serious | Serious | Undetected | 213 | 203 | 0.39  (-0.19; 0.98) | ⨁◯◯◯ |

**Supplementary Material 4**

Pain intensity: Sample size (n), mean, and standard deviation (sd) at post-intervention and follow-up for studies included in the meta-analysis.

| Author | Digital Group | | | Control Group | | | Follow-up period (months) | Digital group | | Control group | |
| --- | --- | --- | --- | --- | --- | --- | --- | --- | --- | --- | --- |
|  | n | Mean | SD | n | Mean | SD |  | Mean | SD | Mean | SD |
| Weber et al 2024 | 32 | -68,30 | 16,10 | 28 | -55,50 | 22,50 |  |  |  |  |  |
| Moutzouri et al 2024 | 22 | 3,40 | 0,80 | 22 | 4,00 | 1,20 | 3 | 2,40 | 1,30 | 3,20 | 1,10 |
| Master et al 2024 | 8 | 1,90 | 1,90 | 8 | 1,50 | 1,40 | 6 | 2,40 | 2,00 | 1,60 | 1,00 |
| Zhao et al 2024 | 50 | 1,90 | 1,50 | 50 | 1,70 | 1,40 |  |  |  |  |  |
| Shim et al 2022 | 27 | 5,10 | 2,00 | 27 | 4,70 | 2,00 | 12 | 2,80 | 1,50 | 3,30 | 2,10 |
| Lee et al 2023 | 15 | 33,31 | 18,87 | 16 | 34,80 | 20,61 |  |  |  |  |  |
| Thiengwittayaporn et al. 2023 | 42 | -73,30 | 7,20 | 20 | -70,70 | 5,90 |  |  |  |  |  |
| Nuevo et al 2024 | 23 | 1,55 | 1,26 | 22 | 2,55 | 2,13 |  |  |  |  |  |
| An et al. 2021 | 18 | 11,00 | 1,14 | 35 | 12,12 | 2,07 | 1,5 |  |  |  |  |
| Fanning et al 2020 | 15 | 54,27 | 8,06 | 13 | 59,79 | 8,06 |  |  |  |  |  |
| Doiron-Cadrin et al 2019 | 12 | 7,80 | 4,00 | 22 | 8,69 | 3,80 |  |  |  |  |  |
| Pronk et al 2020 | 38 | 12,43 | 12,17 | 33 | 13,33 | 15,50 |  |  |  |  |  |
| Pelle et al 2020 | 214 | -59,50 | 16,50 | 213 | -57,40 | 18,00 | 6 | -62,10 | 20,80 | -57,50 | 18,00 |
| Bettger et al 2020 | 151 | -66,60 | 15,60 | 153 | -68,70 | 17,10 | 3 | -82,70 | 13,60 | -76,70 | 17,50 |
| Zadro et al 2019 | 30 | 3,80 | 2,40 | 30 | 4,40 | 2,30 |  |  |  |  |  |
| Kloek et al 2018 | 109 | -55,80 | 39,88 | 99 | -48,80 | 41,76 | 1,5 | -65,90 |  | -61,60 |  |
| Bennell et al 2017 | 74 | 3,30 | 2,20 | 74 | 5,10 | 2,00 | 9 |  |  |  |  |
| Rini et al 2015 | 58 | 4,07 | 1,99 | 55 | 4,62 | 1,79 |  |  |  |  |  |
| Bossen et al 2013 | 100 | 3,50 | 4,87 | 99 | 4,50 | 5,20 | 12 | 3,50 |  | 3,80 |  |
| Duong et al 2023 | 51 | 1,70 | 1,40 | 51 | 2,60 | 2,10 |  |  |  |  |  |
| Charlton et al 2023 | 10 | 2,30 | 1,54 | 10 | 3,00 | 1,98 |  |  |  |  |  |
| Moffet et al 2015 | 104 | 77,20 | 14,28 | 101 | 76,90 | 14,07 | 2 |  |  |  |  |
| Osterloh et al 2023 | 13 | 3,65 | 3,38 | 13 | 5,55 | 3,38 |  |  |  |  |  |
| Kane et al 2020 | 28 | 14,00 | 3,20 | 30 | 14,30 | 3,20 |  |  |  |  |  |
| Nelson et al 2020 | 35 | -83,00 | 14,00 | 35 | -85,00 | 14,00 | 6 | -91,00 | 10,00 | -89,00 | 13,00 |
| Allen et al 2018 | 142 |  |  | 140 |  |  |  |  |  |  |  |
| Janhunen et al 2022 | 21 | 20,80 | 20,30 | 25 | 27,00 | 27,50 |  |  |  |  |  |
| Lo et al 2024 | 15 | 4,78 | 1,77 | 15 | 4,56 | 2,39 |  |  |  |  |  |
| Akgül et al 2025 | 15 | 2,20 | 1,10 | 15 | 3,30 | 1,50 |  |  |  |  |  |

Self-reported disability: Sample size (n), mean, and standard deviation (sd) at post-intervention and follow-up for studies included in the meta-analysis.

| Authors | Digital Group | | | Control group | | | Follow-up period | Digital Group | | Control group | |
| --- | --- | --- | --- | --- | --- | --- | --- | --- | --- | --- | --- |
|  | n | Mean | SD | n | Mean | SD |  | Mean | SD | Mean | SD |
| Weber et al 2024 | 32 | -71,80 | 14,30 | 28 | -63,70 | 23,90 |  |  |  |  |  |
| Barret et al 2024 | 29 | 40,90 | 10,50 | 29 | 39,80 | 7,50 | 12,00 | 42,50 | 9,50 | 40,50 | 7,60 |
| Moutzouri et al 24 | 22 | -66,00 | 18,30 | 22 | -66,00 | 22,00 | 3,00 |  |  |  |  |
| Master et al 24 | 8 | 13,40 | 10,00 | 8 | 5,00 | 4,90 | 6,00 | 10,80 | 13,40 | 5,80 | 9,90 |
| Zhao et al 24 | 50 | 15,10 | 7,30 | 50 | 14,40 | 6,70 |  |  |  |  |  |
| Shim et al 22 | 27 | 66,10 | 12,50 | 27 | 62,20 | 15,00 | 12,00 | 46,50 | 9,10 | 45,30 | 15,00 |
| Thiengwittayaporn et al, 23 | 42 | -80,40 | 9,80 | 20 | -71,20 | 7,00 |  |  |  |  |  |
| Nuevo et al 24 | 23 | 18,49 | 11,51 | 22 | 21,34 | 11,76 |  |  |  |  |  |
| An et al, 21 | 18 | 40,67 | 4,81 | 35 | 48,05 | 5,46 | 1,50 |  |  |  |  |
| Fanning et al 20 | 12 | 63,27 | 5,09 | 13 | 61,67 | 5,09 |  |  |  |  |  |
| Doiron-Cadrin et al 19 | 12 | 26,30 | 9,80 | 22 | 32,26 | 12,62 |  |  |  |  |  |
| Pronk et al 2020 | 38 | 36,50 | 10,50 | 33 | 39,60 | 9,80 |  |  |  |  |  |
| Pelle et al 2020 | 214 | -61,40 | 19,30 | 213 | -58,50 | 19,60 | 6,00 | -62,10 | 20,80 | -58,60 | 19,30 |
| Bettger et al, 2019 | 151 | -76,40 | 13,90 | 153 | -75,70 | 16,60 | 12,00 | -82,70 | 13,60 | -80,90 | 17,70 |
| Zadro et al, 2019 | 30 | 4,90 | 4,50 | 30 | 6,40 | 4,40 |  |  |  |  |  |
| Kloek et al, 2018 | 109 | -66,70 | 18,20 | 99 | -62,20 | 20,40 | 12,00 | -69,30 | 18,70 | -65,30 | 22,80 |
| Bennell et al 2017 | 74 | 18,30 | 10,70 | 74 | 27,60 | 11,70 | 9,00 |  |  |  |  |
| Bossen et al 2013 | 100 | -67,80 | 39,63 | 99 | -61,30 | 38,64 | 12,00 | -67,90 | 44,90 | -62,90 | 44,67 |
| Charlton et al 2023 | 10 | -74,30 | 10,55 | 10 | -77,60 | 12,79 |  |  |  |  |  |
| Moffet et al 2015 | 104 | 80,50 | 13,26 | 101 | 80,30 | 13,07 | 2,00 |  |  |  |  |
| Osterloh et al 2023 | 13 | 13,96 | 13,25 | 13 | 20,54 | 13,25 |  |  |  |  |  |
| Timmers et al, 2019 | 114 | 37,61 | 10,17 | 99 | 43,08 | 12,96 |  |  |  |  |  |
| Crawford et al, 2021 | 208 | -70,40 | 12,60 | 244 | -73,60 | 13,40 |  |  |  |  |  |
| Nelson et al, 2020 | 35 | -83,00 | 14,00 | 35 | -84,00 | 12,00 | 6,00 | -91,00 | 10,00 | -88,00 | 11,00 |
| Rini et al 2015 | 58 | 1,62 | 1,19 | 55 | 1,75 | 1,24 |  |  |  |  |  |
| Janhunen et al 2023 | 21 | 38,60 | 6,10 | 25 | 36,70 | 6,70 |  |  |  |  |  |
| Lo et al 2024 | 15 | 4,78 | 1,77 | 15 | 4,56 | 2,39 |  |  |  |  |  |
| Akgül et al 2025 | 15 | 15,10 | 7,90 | 15 | 24,70 | 10,10 |  |  |  |  |  |

Performance: Sample size (n), mean, and standard deviation (sd) at post-intervention and follow-up for studies included in the meta-analysis.

|  | Digital group | | | Control group | | |
| --- | --- | --- | --- | --- | --- | --- |
| Authors | n | Mean | SD | n | Mean | SD |
| Weber et al 2024 | 32 | -16,60 | 3,50 | 28 | -17,50 | 6,40 |
| Barret et al 2024 | 29 | -19,30 | 9,10 | 29 | -19,20 | 7,90 |
| Moutzouri et al 24 | 22 | 9,30 | 1,30 | 22 | 10,40 | 2,00 |
| Zhao et al 24 | 50 | 17,70 | 4,30 | 50 | 19,40 | 3,50 |
| Shim et al 22 | 27 | -0,53 | 0,22 | 27 | -0,61 | 0,30 |
| Lee et al 23 | 15 | 7,80 | 1,00 | 16 | 8,04 | 0,81 |
| Nuevo et al 24 | 23 | 13,74 | 4,22 | 22 | 15,94 | 10,02 |
| An et al, 21 | 18 | 10,61 | 1,26 | 35 | 12,77 | 1,87 |
| Fanning et al 20 | 12 | -10,20 | 1,20 | 13 | -9,58 | 1,20 |
| Doiron-Cadrin et al 19 | 12 | 8,50 | 1,40 | 22 | 11,23 | 3,69 |
| Bettger et al, 2019 | 151 | -1,00 | 0,30 | 153 | -1,00 | 0,30 |
| Kloek et al, 2018 | 109 | 7,30 | 1,70 | 99 | 7,30 | 2,40 |
| Charlton et al 2023 | 10 | -1,45 | 0,16 | 10 | -1,32 | 0,15 |
| Moffet et al 2015 | 104 | 30,60 | 13,26 | 101 | 34,20 | 13,07 |
| Osterloh et al 2023 | 13 | 8,05 | 3,16 | 13 | 9,88 | 3,20 |
| Crawford et al, 2021 | 208 | 9,30 | 3,30 | 244 | 10,10 | 4,80 |
| Nelson et al, 2020 | 35 | 9,50 | 2,60 | 35 | 11,60 | 4,40 |
| Janhunen et al 2023 | 21 | 7,60 | 1,50 | 25 | 7,70 | 1,20 |

**Supplementary Material 5 – Forest plots of the meta-analysis**

Pain intensity (chronic conditions); comparison: no intervention


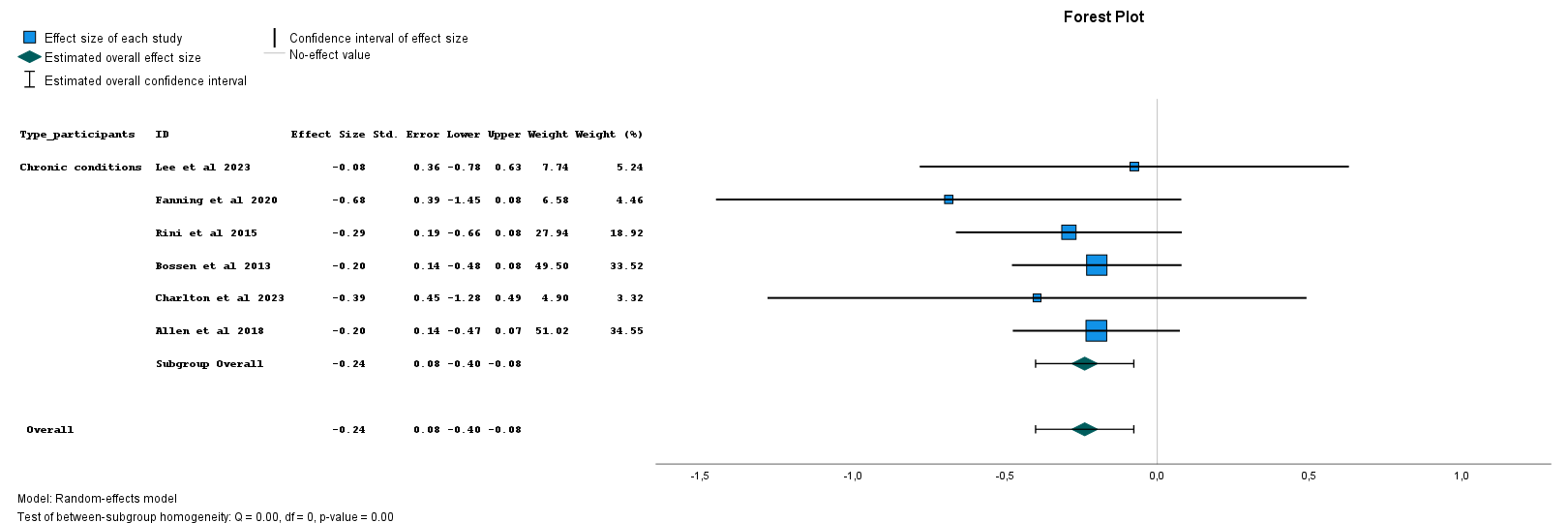


Sensitivity analysis: pain intensity; studies using none or 1 personalization strategy


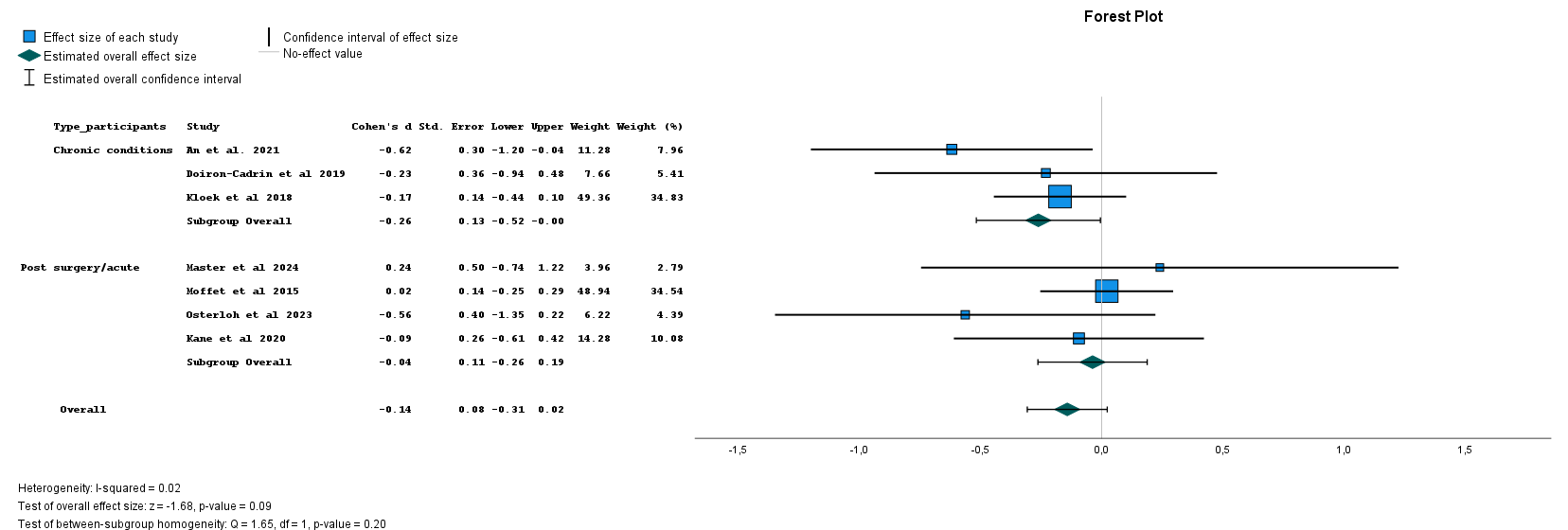


Sensitivity analysis: pain intensity; studies using asynchronous digital interventions (comparison: other interventions)


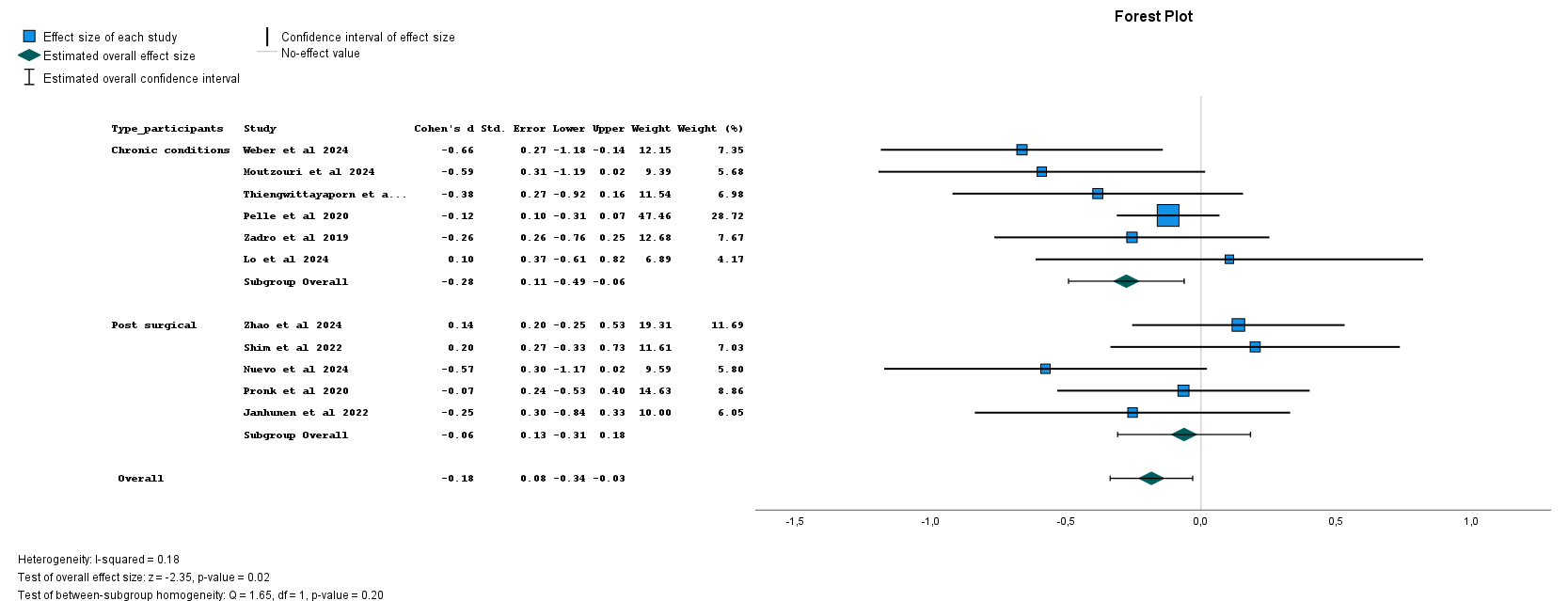


Self-reported disability (chronic conditions); comparison: no intervention


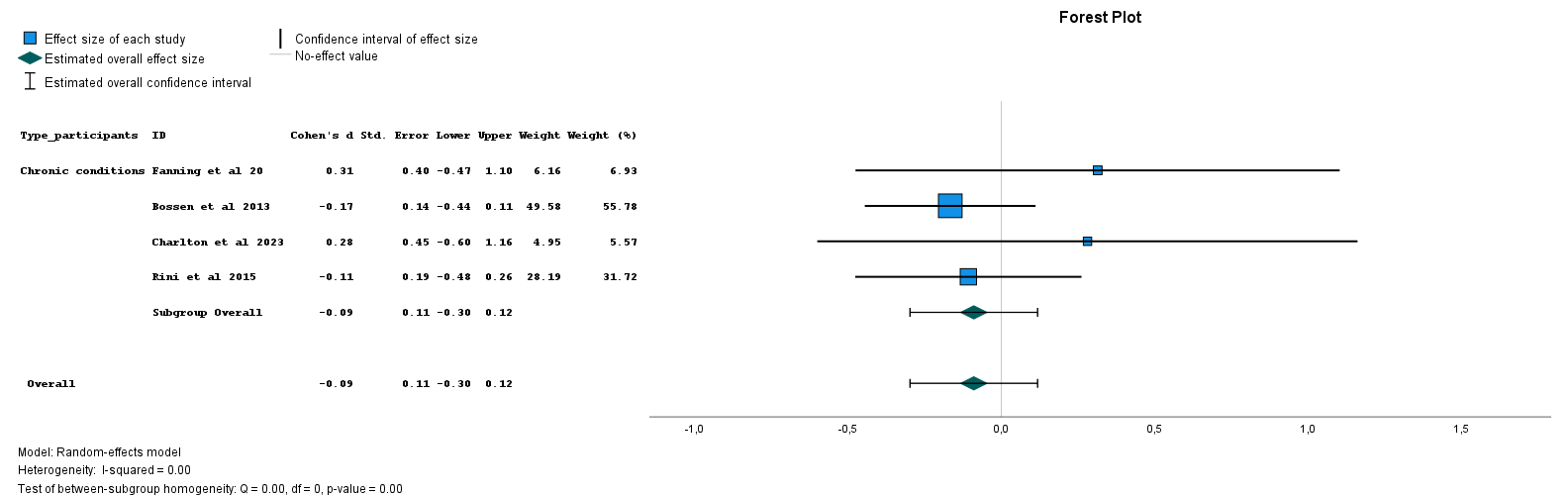


Sensitivity analysis: self-reported disability; studies using none or 1 personalization strategy


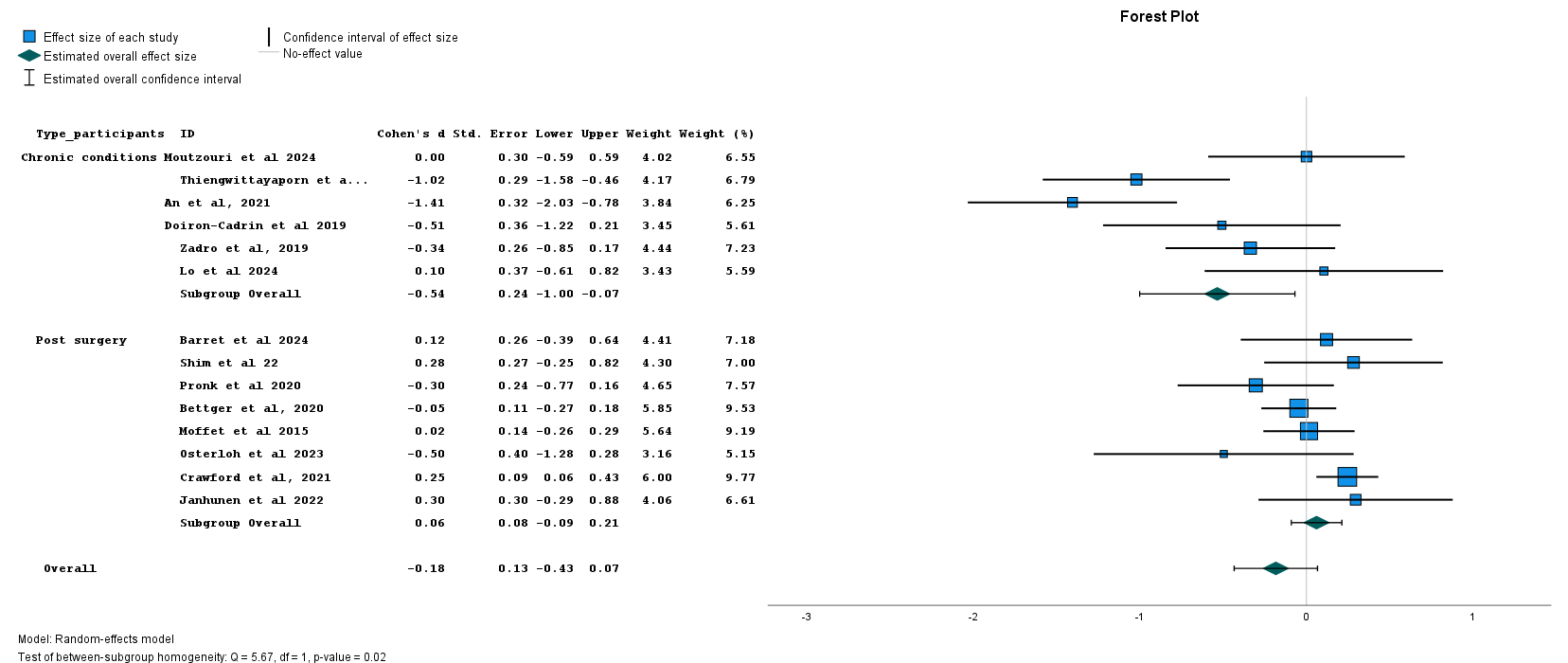


Sensitivity analysis: self-reported disability; studies using asynchronous digital interventions (comparison: other interventions)


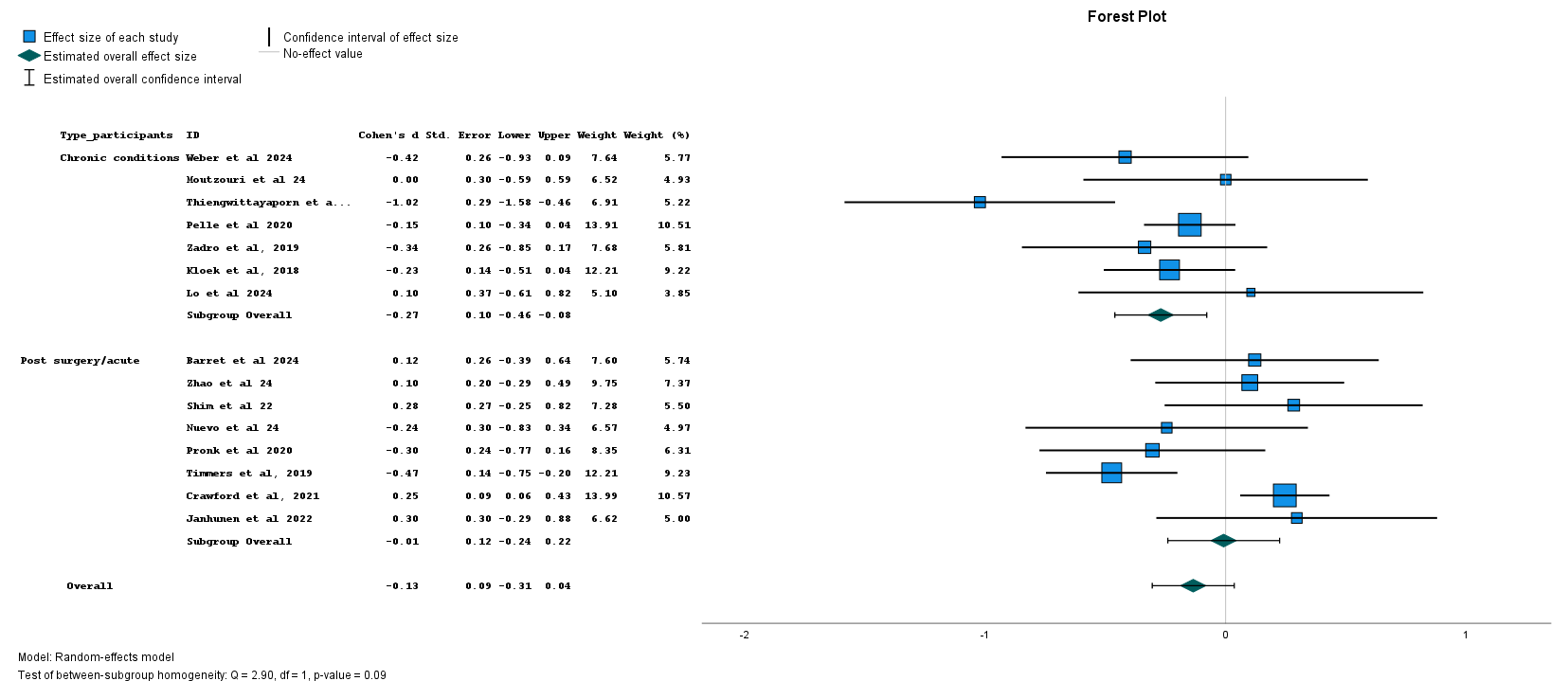


Performance

Performance; comparison: no intervention


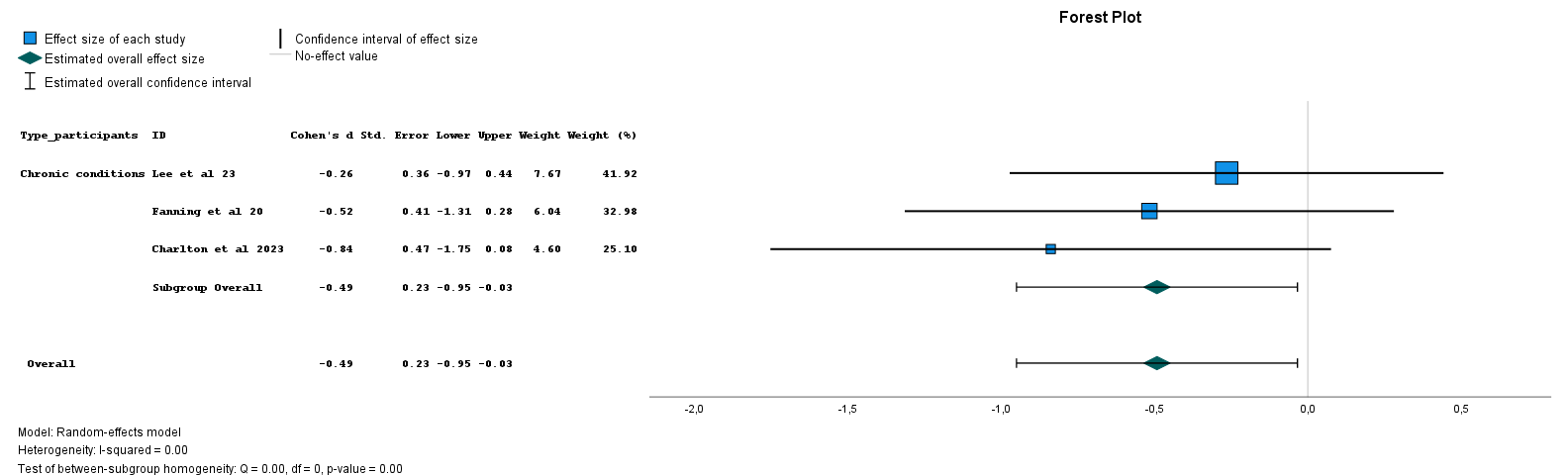


Sensitivity analysis: performance; studies using none or 1 personalization strategy


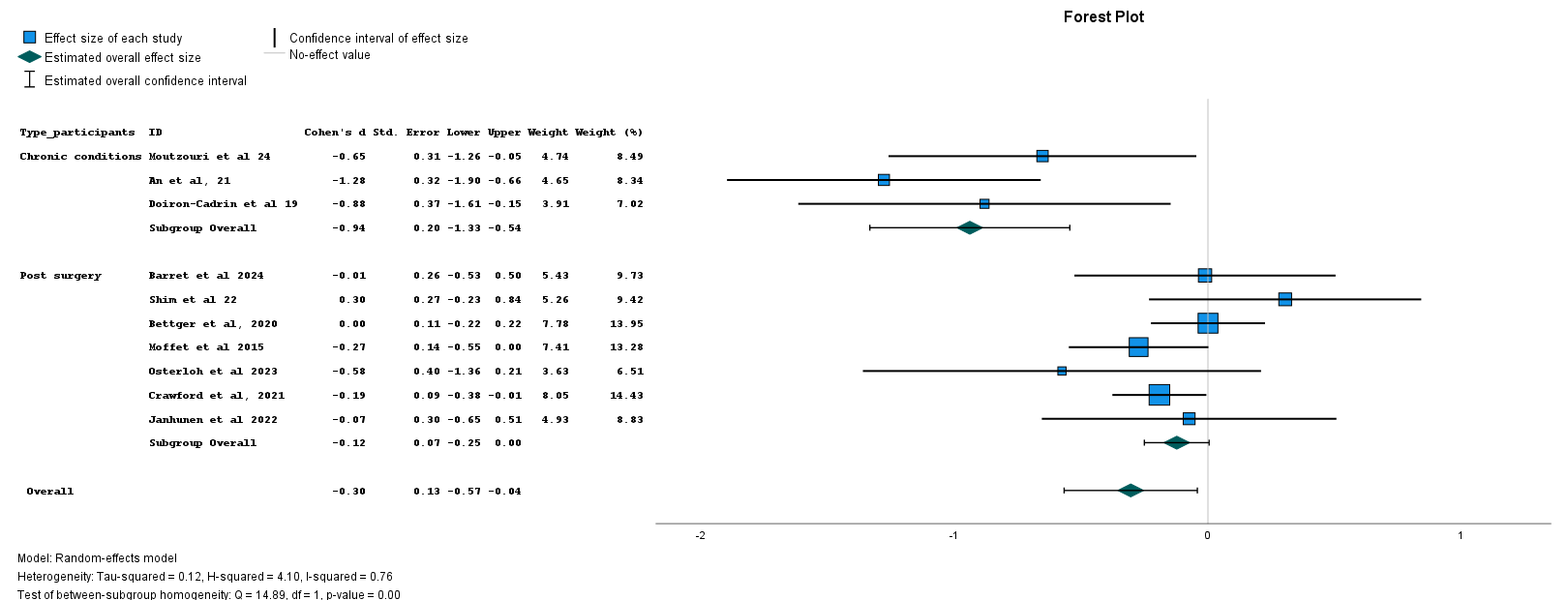


Sensitivity analysis: performance; studies using asynchronous digital interventions (comparison: other interventions)


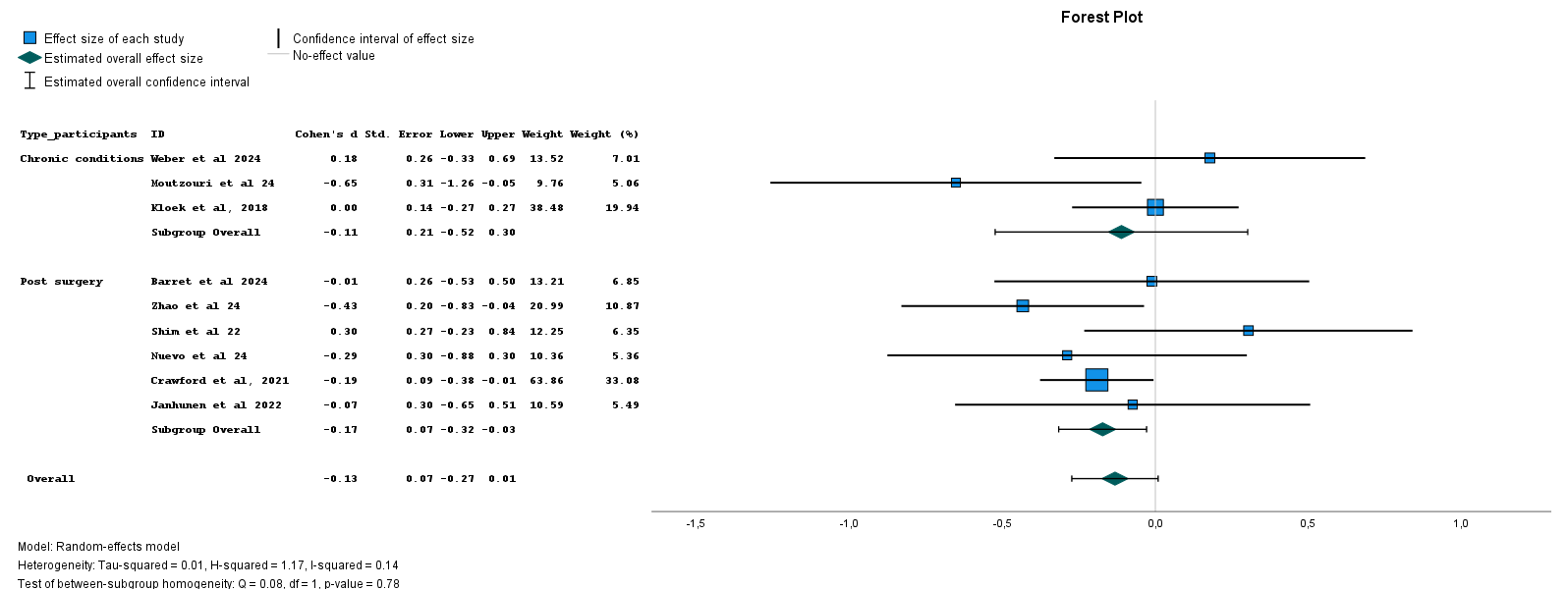


Psychological variables

Self-efficacy Performance; comparison: other interventions


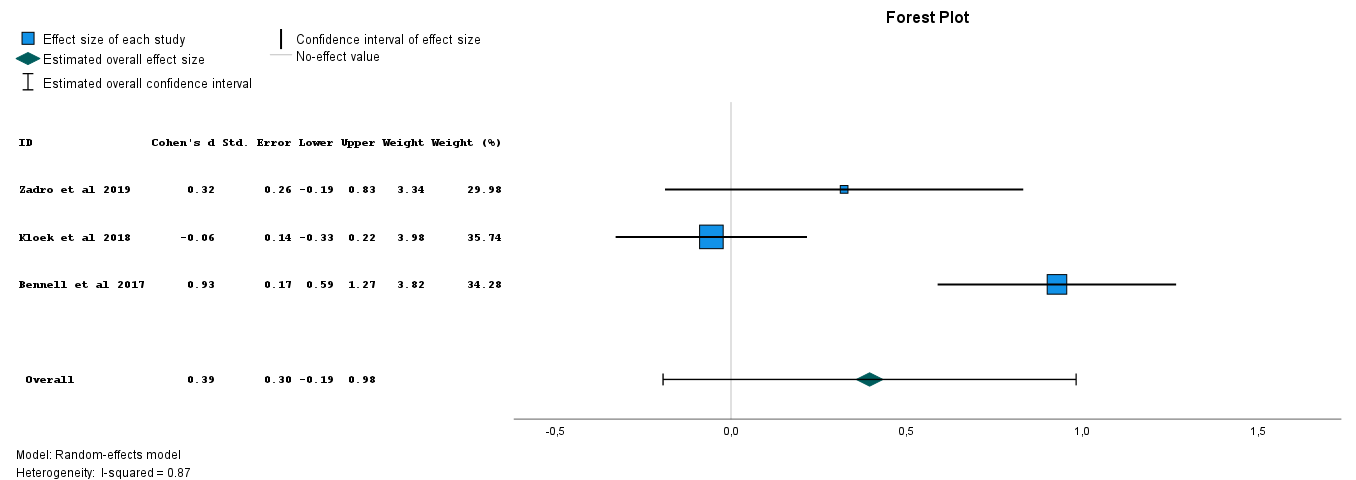


**Supplementary material 6 – Qualitative analysis of studies not included in the meta-analysis**

Pain intensity

Three studies were not included in the meta-analysis and compared a digital intervention against other interventions [8][6] or a sham digital intervention [9]. One study included participants with hand osteoarthrosis [8], another study used participants with knee or hip osteoarthrosis [6] and another included participants who were submitted to knee arthroplasty [9]. All studies found a between-group significant difference, consistent with a decrease in pain intensity, that favoured the digital intervention at post-intervention [6,8,9] and at 6-month follow-up [8].

Self-reported disability

Five studies were not included in the meta-analysis and compared a digital intervention against other interventions [5,6,8,10] or a sham digital intervention [9]. One study included participants with hand osteoarthrosis [8], another included participants with knee or hip osteoarthrosis [6] and three participants who were submitted to knee arthroplasty [5,9,10] All studies found a between-group significant difference, consistent with a decrease in self-reported disability, that favoured the digital intervention at post-intervention [5,6,8–10], 1R), at 6 months follow-up [8] and at 12-month follow-up [5].

Performance

Four studies were not included in the meta-analysis [2,6,8,10]. These studies compared a digital intervention against other interventions and used participants with hand osteoarthritis [8], knee or hip osteoarthritis [2,6] and knee arthroplasty [10]. One study reported an improvement in performance favouring the digital intervention group [6], while the remaining three reported no difference between the group receiving a digital intervention and the group receiving another intervention [2,8,10], nor between the group receiving the digital intervention and a wait-list control group [2].

Psychological variables

Self-efficacy

Two studies assessed self-efficacy at post-intervention [4,7], one of which also assessed it at 12-month follow-up. One study used participants with Knee or hip osteoarthrosis and compared a digital intervention against no intervention and reported no between-group differences [7]. The other study [4], compared a digital intervention against a waiting list control and reported between-group differences favouring the digital intervention for patients with knee and hip osteoarthrosis at post-intervention, but no between-group differences at 12-month follow-up.

Catastrophizing

Two studies assessed catastrophizing at post-intervention [1,3] against other interventions and for patients with knee pathology. One study [3] reported a decreased pain catastrophizing, favouring the digital group, while the other [1] reported no between-group differences.

Fear of movement

One study [11] assessed fear of movement in participants with low back pain and reported no differences at post-intervention between the group receiving a digital intervention and a group performing usual activities.

Anxiety

One study [7] assessed fear of movement in participants with hip or knee osteoarthritis and reported no differences at post-intervention between the group receiving a digital intervention and a group receiving no intervention.

References

[1] Akgül H, Birtane M, Tonga E. Effects of a Digitally Supported Physical Activity Intervention in Knee Osteoarthritis: A Pilot Randomized Controlled Trial. Musculoskeletal Care 2025;23:1–12.

[2] Allen KD, Arbeeva L, Callahan LF, Golightly YM, Goode AP, Heiderscheit BC, Huffman KM, Severson HH, Schwartz TA. Physical therapy vs internet-based exercise training for patients with knee osteoarthritis: results of a randomized controlled trial. Osteoarthr Cartil 2018;26:383–396.

[3] Bennell KL, Nelligan R, Dobson F, Rini C, Keefe F, Kasza J, French S, Bryant C, Dalwood A, Abbott JH, Hinman RS. Effectiveness of an internet-delivered exercise and pain-coping skills training intervention for persons with chronic knee pain: A randomized trial . Ann Intern Med 2017;166:453–462. doi:10.7326/M16-1714.

[4] Bossen D, Veenhof C, Van Beek KE, Spreeuwenberg PM, Dekker J, De Bakker DH. Effectiveness of a Web-Based Physical Activity Intervention in Patients With Knee and/or Hip Osteoarthritis: Randomized Controlled Trial. J Med Internet Res 2013;15:e257. doi:10.2196/jmir.2662.

[5] Duong V, Robbins SR, Dennis S, Venkatesha V, Ferreira ML, Hunter DJ. Combined Digital Interventions for Pain Reduction in Patients Undergoing Knee Replacement. JAMA Netw OPEN 2023;6.

[6] Gohir SA, Eek F, Kelly A, Abhishek A, Valdes AM. Effectiveness of Internet-Based Exercises Aimed at Treating Knee Osteoarthritis The iBEAT-OA Randomized Clinical Trial. JAMA Netw OPEN 2021;4.

[7] Rini C, Porter LS, Somers TJ, McKee DC, DeVellis RF, Smith M, Winkel G, Ahern DK, Goldman R, Stiller JL, Mariani C, Patterson C, Jordan JM, Caldwell DS, Keefe FJ. Automated Internet-based pain coping skills training to manage osteoarthritis pain: A randomized controlled trial . Pain 2015;156:837–848. doi:10.1097/j.pain.0000000000000121.

[8] Sánchez-Laulhé PR, Biscarri-Carbonero Á, Suero-Pineda A, Luque-Romero LG, Barrerogarcía FJ, Blanquero J, Heredia-Rizo AM. The effects of a mobile app-delivered intervention in people with symptomatic hand osteoarthritis: a pragmatic randomized controlled trial . Eur J Phys Rehabil Med 2023;59:54–64. doi:10.23736/S1973-9087.22.07744-9.

[9] Timmers T, Janssen L, van der Weegen W, Das D, Marijnissen WJ, Hannink G, van der Zwaard BC, Plat A, Thomassen B, Swen JW, Kool RB, Heerspink FOL. The effect of an app for day-to-day postoperative care education on patients with total knee replacement: Randomized controlled trial. JMIR mHealth uHealth 2019;7:1–16.

[10] Tousignant M, Moffet H, Boissy P, Corriveau H, Cabana F, Marquis F. A randomized controlled trial of home telerehabilitation for post-knee arthroplasty. J Telemed Telecare 2011;17:195–198.

[11] Zadro JR, Shirley D, Simic M, Mousavi SJ, Ceprnja D, Maka K, Sung J, Ferreira P. Video-Game-Based Exercises for Older People with Chronic Low Back Pain: A Randomized Controlledtable Trial (GAMEBACK) . Phys Ther 2019;99:14–27. doi:10.1093/ptj/pzy112.
